# In situ supramolecular polymerization-enhanced self-assembly of polymer vesicles for highly efficient photothermal therapy

**DOI:** 10.1038/s41467-020-15427-1

**Published:** 2020-04-07

**Authors:** Yannan Liu, Hao Wang, Shanlong Li, Chuanshuang Chen, Li Xu, Ping Huang, Feng Liu, Yue Su, Meiwei Qi, Chunyang Yu, Yongfeng Zhou

**Affiliations:** 1 School of Chemistry and Chemical Engineering, Frontiers Science Center for Transformative Molecules, Shanghai Jiao Tong University, 800 Dongchuan Road, 200240 Shanghai, P. R. China; 2 Key Laboratory of Optoelectronic Devices and Systems of Ministry of Education, Shenzhen University, 518060 Shenzhen, P. R. China; 3 Joint Research Center for Precision Medicine, Shanghai Jiao Tong University Affiliated Sixth People’s Hospital South Campus, 6600th Nanfeng Road, Fengxian District, 201499 Shanghai, P. R. China

**Keywords:** Supramolecular polymers, Self-assembly, Drug delivery

## Abstract

Vesicular photothermal therapy agents (PTAs) are highly desirable in photothermal therapy (PTT) for their excellent light-harvesting ability and versatile hollow compartments. However, up to now, the reported vesicular PTAs are generally self-assembled from small molecules like liposomes, and polymer vesicles have seldom been used as PTAs due to the unsatisfactory photothermal conversion efficiency resulting from the irregular packing of chromophores in the vesicle membranes. Here we report a nano-sized polymer vesicle from hyperbranched polyporphyrins with favorable photothermal stability and extraordinarily high photothermal efficiency (44.1%), showing great potential in imaging-guided PTT for tumors through in vitro and in vivo experiments. These excellent properties are attributed to the in situ supramolecular polymerization of porphyrin units inside the vesicle membrane into well-organized 1D monofilaments driven by π–π stacking. We believe the supramolecular polymerization-enhanced self-assembly process reported here will shed a new light on the design of supramolecular materials with new structures and functions.

## Abstract (web summary)

Photothermal therapy (PTT) has recently emerged as a promising approach for cancer therapy. Here, the authors report a hyperbranched polymer vesicle with favorable photothermal stability and high photothermal efficiency for PTT through a supramolecular polymerization-enhanced self-assembly strategy.

## Introduction

PTT is promising for cancer therapy and skin treatment due to its advantages of spatially targeted light irradiation, minimal invasiveness, and reducing patients’ pain^1–3^. Up to now, both inorganic and organic nanomaterials have been used as PTAs for PTT studies^4–7^. Organic PTAs can be divided into two categories in view of the structure: one is solid organic PTAs such as carbon nanomaterials^8–10^, conducting polymer nanoparticles^11–13^, black phosphorus^14,15^, nanodots^16^, and so on, the other ones are hollow organic PTAs like vesicles^17,18^. Among them, vesicular PTAs have received considerable attention due to their superior light-harvesting ability resulting from multiple reflections of incident light in hollow compartments of vesicles^19,20^, and prominent versatility by trapping hydrophilic functional units in their hollow cavities as well as hydrophobic units in the membrane. Zheng et al. are pioneered in the vesicular PTAs self-assembled from porphyrin lipids^21–23^. However, up to now, most of the vesicular PTAs are limited to liposomes. The stability of liposomes is not good enough and should be enhanced by the addition of cholesterol or else for further application in PTT^24,25^. Thus, it is highly challenging to develop vesicular PTAs with both stable structure and high photothermal conversion ability.

Polymer vesicles feature not only hollow lumens but also stable and designable membranes. Thus polymer vesicles should be very potential in PTT application. However, to our great surprise, hitherto, it is still very rare to use polymer vesicles as PTAs. The main challenge lies in the poor photothermal conversion efficiency for polymer vesicles^26^. Generally, the membrane fluidity of liposomes is much higher than that of polymer vesicles. Thus, the chromophores inside liposomes can move freely to reach a well-organized π–π stacking state, which leads to a high conversion of harvested light into heat energy. On the contrary, the polymer vesicles are too tough to realize the desirable organization of chromophores, and thus result in a lower photothermal conversion efficiency^17,22^.

To meet the challenge, herein, we develop a novel polymer vesicle with both favorable stability and high photothermal conversion efficiency. In recent years, our groups have reported a serious of polymer vesicles with controlled size from the self-assembly of hyperbranched polymers (HBPs)^27,28^. Most importantly, the HBP vesicles show combined advantages of block copolymer vesicles and liposomes; for example, they have robust membrane structures like traditional block copolymer vesicles and excellent membrane fluidity like liposomes^29,30^. In the present work, we prepare a nano-sized polymer vesicle from the self-assembly of a hyperbranched polyporphyrin. Porphyrins are chosen as model molecules owing to their unique large π conjugated structure and excellent light-harvesting ability^31^. Due to the inherent good fluidity of the HBP vesicles, the porphyrin units inside the vesicle membranes can tune the conformation in a well-organized way through directional π–π stacking. As a result, abundant one-dimensional (1D) supramolecular polymers consisting of linearly staked porphyrin units, like polyporphyrin nanofilaments, are formed inside the vesicle membranes. The vesicles enhanced by supramolecular polyporphyrins inside show unexpectedly high photothermal conversion effect of 44.1% in vitro and satisfactory physiological and photothermal stability. To our knowledge, it is the first report on the vesicles generated through the combination of covalent polymer self-assembly and supramolecular polymerization. Thus we define it as a “supramolecular polymerization-enhanced self-assembly” (SPESA) process. Moreover, the obtained hyperbranched polyporphyrin nanovesicles present excellent biocompatibility and PTT efficiency at the cell level and in vivo. Besides, the vesicles can be facilely functionalized through the encapsulation of functional molecules such as Rhodamine b (Rb) and Cy 7.5, which endow the vesicles with fluorescence monitoring and imaging-guided properties.

## Results

### Preparation and characterization of THPG polymer vesicles

**Fig. 1:**
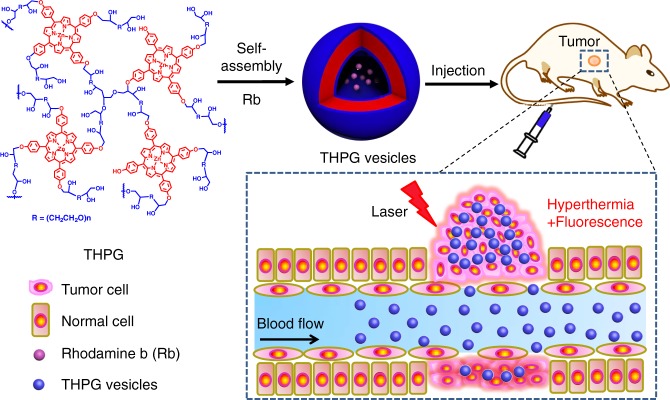
A schematic diagram of hyperbranched polyporphyrin vesicles for photothermal therapy. Nano-sized polymer vesicles from the self-assembly of hyperbranched polyporphyrins show effective photothermal killing of tumors in vivo.

We firstly synthesized a new amphiphilic hyperbranched polyporphyrin. To date, the reports on hyperbranched multi-porphyrin polymers are somewhat limited because of the challenge in synthesis. Previously, Frechet et al.^32^ prepared a hyperbranched polyporphyrin through an A_2_ + B_3_ proton-transfer polymerization. Inspired by them, herein we employed an anion-initiated A_2_ + B_4_ ring-opening polymerization method to prepare amphiphilic hyperbranched polyporphyrins named as THPG (Fig. 1) by using di-epoxy ether as an A_2_ monomer and meso-tetrakis (4-hydroxyphenyl) zinc porphyrin (ZnTHPP) as a B_4_ monomer (Supplementary Fig. 1). It should be noted that a very slow adding of the ZnTHPP to di-epoxy ether solution and keeping low solid content is crucial to avoid gelation during the polymerization. As shown in Supplementary Figs. 2,  3, the structures of ZnTHPP and THPG polymers were confirmed by proton nuclear magnetic resonance (^1^H NMR) spectra. The as-prepared polymers have a number-average molecular weight (Mn) of 6.4 kDa and molecular weight distribution of 1.8 from the GPC test by using THF as the eluent (Supplementary Fig. 4). We also synthesized other THPGs with the feeding ratio between di-epoxy ether and ZnTHPP of 1:1. As expected, the obtained THPG_11_ polymers have more unreacted phenolic hydroxyl groups according to the ^1^H NMR spectrum (Supplementary Fig. 5).

**Fig. 2:**
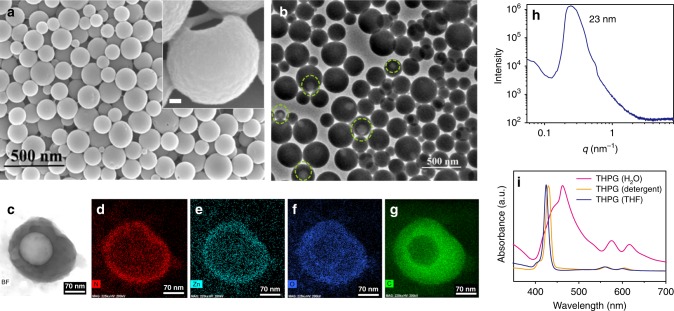
Characterizations of THPG vesicles. **a** The representative SEM image of THPG vesicles. The scale bar in the inset is 30 nm. **b** The representative TEM image of THPG vesicles, light green circles indicated the broken particles featured with bright openings. Similar SEM and TEM images of THPG were obtained for more than three times experiments. **c** The representative STEM image and corresponding elemental mapping images of nitrogen (**d**), zinc (**e**), oxygen (**f**), and carbon (**g**) elements. **h** The SAXS spectrum of THPG vesicle solution at a concentration of 0.5 mg mL^−1^. **i** The UV–Vis spectra of THPGs in water, THF or 5% Triton X-100 detergent aqueous solution, respectively.

The self-assembly of THPGs was induced by dropwise adding the selective solvent of ultrapure H_2_O into the polymer/dimethyl-sulfoxide (DMSO) solution, followed by dialysis against deionized water to remove DMSO. The scanning electron microscopy (SEM) measurement shows that the supramolecular aggregates of THPGs are spherical nanoparticles with an average diameter of 127 nm (Fig. 2a), which is smaller than the hydrodynamic diameter of 176 nm acquired from the dynamic light scattering (DLS) measurement (Supplementary Fig. 6). It is probably due to the shrinkage of the aggregates during the drying process in the SEM measurements. Fortunately, some of the nanoparticles were broken to show the hollow lumens, which indicates the particles are polymer vesicles rather than spherical micelles (inset of Fig. 2a and Supplementary Fig. 7). The hollow vesicular structure was also proved by the transmission electron microscopy (TEM) measurements according to the broken particles featured with bright openings (light green circles in Fig. 2b). The scanning TEM (STEM) measurements of a typically broken particle provided further evidence (Fig. 2c). Nitrogen and zinc elements derived from ZnTHPP units in the particle are not abundant, and the corresponding elemental mapping images (Fig. 2d, e) show the typical vesicular skin/pool structure. While the oxygen and carbon elements are more abundant, and the hollow lumen of the vesicle from the broken opening can be seen clearly (Fig. 2f, g). Thus, THPG vesicles were formed confessedly according to these characterizations. Moreover, the average wall thickness of the vesicles measured from the SEM images is ca. 21 nm. This value is much larger than one bilayer thickness (ca. 3 nm), indicating THPG vesicles should have a multi-lamellar structure. The vesicle wall thickness is about 23 nm according to the Bragg equation of *d* = 2*π* *q*^−1^ determined by the small-angle X-ray scattering (SAXS) experiment (Fig. 2h)^33,34^, which agrees well with the SEM results. The self-assembly of THPG_11_ also generated spherical vesicles according to the TEM image (Supplementary Fig. 8). However, the vesicle size is around 500 nm and is much larger than that of THPG vesicles. It is because more epoxy ether units in THPGs afford higher hydrophilicity compared with THPGs_11_, leading to a smaller vesicle size.

The driving force for the self-assembly of THPG vesicles was explored. Considering the amphiphilic nature of THPGs, long-range hydrophobic interactions should play a significant role in the vesicle formation. Besides, the π–π stacking interaction is also important for the self-assembly process. As showed in Fig. 2i, the ultraviolet–visible (UV–Vis) absorption spectrum of THPG unimers in tetrahydrofuran (THF, a good solvent) exhibits two strong absorption regions assigned to B band (425 nm) and Q band (from 545 to 626 nm) of porphyrins. However, the absorption peak of THPG vesicles in water was significantly broadened, the B band markedly red-shifted to 463 nm, and the Q band extended to longer wavelength ranging from 560 to 650 nm. It is very rare to observe such a large redshift for most of the porphyrin self-assemblies, which suggests the formation of strong J-aggregates due to the π–π stacking among porphyrins in THPG vesicles. Besides, we also used 5% Triton X-100 as an aqueous detergent solvent for THPG vesicles^18^. As a result, the J-aggregate peak in 463 nm disappeared and the peaks of THPG unimers appeared again in UV–Vis spectra (Fig. 2i), indicating the importance of π–π stacking for keeping the vesicle structure. In addition, it also suggests that there is only the supramolecular interaction in the THPG vesicles without chemical cross-linking, and vesicles can disassemble into unimers in the presence of Triton X-100 aqueous detergents.

**Fig. 3:**
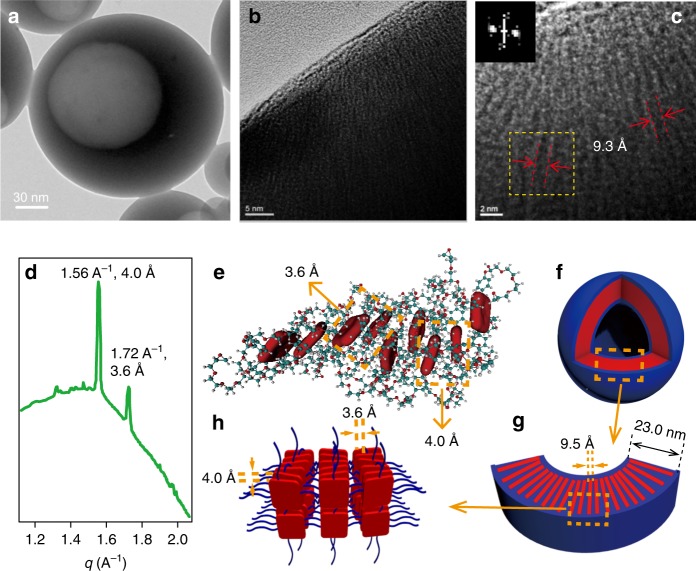
Proposed membrane structure of THPG vesicles. A representative HR-TEM image (**a**) and the magnified ones (**b**, **c**) of a single broken THPG vesicle. The inset in **c** is the fast Fourier transform (FFT) image of selected yellow square area in **c**. Similar HR-TEM images of THPG were observed for more than three separated samples. **d** Synchrotron SAXS of THPG vesicles. **e** The packing model of the porphyrin moieties in THPG vesicles from MD simulation. The red squares in **e** represent the ZnTHPP moieties. **f** A diagrammatic drawing of a THPG vesicle. **g** The magnified membrane cross-section. **h** The packing model of THPGs in the vesicle membrane.

The molecular arrangement mode in the membrane of THPG vesicles was also carefully studied. A typical broken vesicle was selected for high-resolution TEM (HR-TEM) measurements (Fig. 3a), and the marked black nanofilaments and white stripes were observed from the enlarged HR-TEM images of the cross-section of the vesicle membrane (Fig. 3b, c). Considering the electron density difference, the black nanofilaments should result from the supramolecular polymerization of hydrophobic porphyrin units through π–π interaction, while the white stripes should derive from hydrophilic di-epoxy ether moieties. The orientation arrangement of the nanofilaments in the vesicles was further proved by two evident diffraction points shown in the fast Fourier transform (FFT) image (Inset in Fig. 3c) deduced from the selected small yellow square area in Fig. 3c. The average width of the black nanofilaments was measured to be ca. 9.3 Å from the HR-TEM images (Fig. 3c). Meanwhile, a peak in 9.2° with a corresponding d-space of 9.5 Å was observed from the XRD spectrum of THPG vesicles (Supplementary Fig. 9), which agrees well with HR-TEM observation to support the formation of well-organized porphyrin nanofilaments in the vesicles.

The above-mentioned results indicate that porphyrin units spontaneously supra-polymerize into nanofilaments with a thickness around 9.5 Å through directional π–π stacking. However, this thickness is a little bit smaller than the width of the ZnTHPP molecules (1.08 nm in length of side and 1.56 nm in diagonal length from a 3D molecular structure in Supplementary Fig. 10a). Thus, it is not possible for ZnTHPP molecules to pack in the form of H aggregates (Supplementary Fig. 10b). On the contrary, there should be some inclined arrangement with a tilt angle among ZnTHPP units in the nanofilaments as shown Supplementary Fig. 10c, supporting the J-aggregation process. As further evidence, the synchrotron SAXS measurement (Fig. 3d) shows two sharp peaks at *q* = 1.72 and 1.56 Å^−1^ in the spectrum, which can be attributed to the π–π stacking between adjacent porphyrin units with corresponding distances of 3.6 and 4.0 Å according to Bragg equation, respectively. The distance of 3.6 Å is generally assigned to a typical face to face paralleled arrangement among porphyrins, while the distance 4.0 Å should originate from the non-paralleled porphyrin pairs with a certain angle of tilt. Moreover, the circular dichroism (CD) spectrum of THPG vesicles in solid state shows weak chiral signal (Supplementary Fig. 11), which also suggests the porphyrins should be supra-polymerized into nanofilaments in the vesicles with a slight spiral twist structure.

A molecular dynamics (MD) simulation was further carried out to prove the self-assembly mechanism. Here we constructed a simplified model of THPG molecule with one porphyrin linked four arms (Supplementary Fig. 12), and put nine of them in a water box sized 8.0 × 8.0 × 8.0 nm^3^. The self-assembly structure in Fig. 3e shows that porphyrin moieties are apt to ordered arrangement through π–π stacking. Moreover, the porphyrin moieties took a certain spiral arrangement with a tilt angle in chain direction, and the intermolecular distance between two porphyrin units was measured to be 3.6 Å for parallel arrangement and 4.5 Å for titled arrangement. The simulation results support the experimental data well, and the average energy of each porphyrin pairs due to the π–π stacking was calculated to be ca. 29.7 kCal mol^−1^, which is comparable with that of other porphyrin aggregates^16^.

Combining all these experimental and simulation results, the self-assembly of THPG vesicles can be summarized as follows. THPGs first self-assembled into vesicles with membrane thickness ca. 23 nm (Fig. 3f, g) in water driven by hydrophobic interactions. Such a covalent polymer self-assembly process triggered the close aggregation of intra- or inter-molecular porphyrin groups, which prompted the supramolecular polymerization of porphyrin groups into nanofilaments (Fig. 3g) with a little helical J-aggregation driven by π–π stacking interactions (Fig. 3h). The average width of porphyrin nanofilaments is ca. 9.5 Å. Thus, the THPG vesicles are greatly enhanced by the supramolecular polymerization of porphyrin units inside the membranes. To our knowledge, such a unique vesicle structure induced by the synergistic covalent and noncovalent polymer self-assembly process has not been reported so far.

**Fig. 4:**
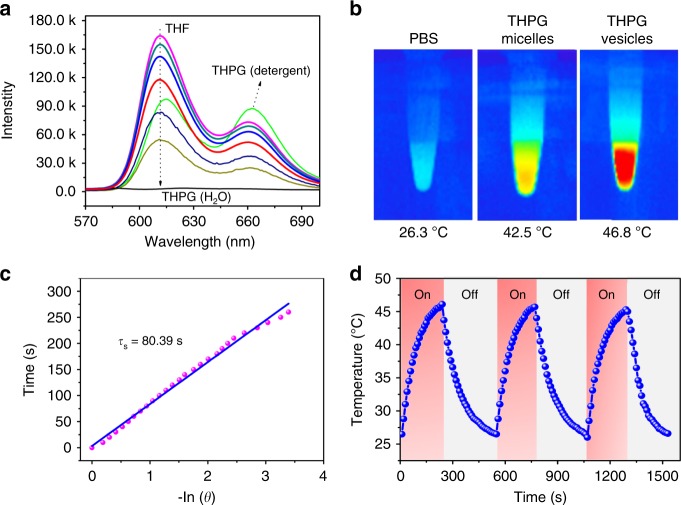
Photothermal efficiency and stability of THPG vesicles. **a** Fluorescence spectra of THPGs in various THF/water solutions and 5% Triton X-100 detergent solution. **b** Solutions of PBS, 0.3 mg mL^−1^ THPG micelles and 0.3 mg mL^−1^ THPG vesicles irradiated with 635 nm laser (200 mW cm^2^) and imaged with a thermal camera. **c** Linear fitting curve of the cooling time of THPG vesicles as a function of negative logarithm of temperatures (*τ*_s_ is the time constant of the sample system). **d** Photothermal stability evaluation of THPG vesicles exposed under 635 nm laser (200 mW cm^−2^) in water.

The photoluminescence performance of THPG vesicles was studied as well. THPG polymers showed strong fluorescence in THF, however, it decayed gradually with the addition of water until totally quenched in pure water (Fig. 4a). As mentioned above, the addition of water into the THPG/THF solution triggered the self-assembly of THPG vesicles. As a result, the supramolecular polymerization of porphyrin moieties driven by π–π stacking interactions happened to form nanofilaments inside the vesicles, leading to the strong fluorescence self-quenching. As further evidence, the fluorescence showed a significant recovery (Fig. 4a) with the addition of 5% Triton X-100 as aqueous detergents into THPG vesicles, which was induced by the disassembly of vesicles into unimers due to the disruption of π–π stacking inside the vesicles. The self-quenching ratio reached up to ca. 1100, which is higher than the value of most porphyrin assemblies^21^. Generally, dyes with strong self-quenching are able to produce good photothermal conversion as proposed by Zheng et al.^22^, and thus THPG vesicles might be promising as efficient PTAs.

To verify the photothermal property of THPG vesicles, the temperature changes of aqueous vesicle solution (0.3 mg mL^−1^) irradiated under 635 nm laser were recorded by an infrared (IR) thermal mapping apparatus, and PBS solution was served as the control. As expected, a rapid temperature rise from 24.1 °C to 46.8 °C was observed for the THPG vesicle solution even at a very low laser power density of 200 mW cm^−2^, while the temperature of PBS solution only rose slightly to 26.3 °C under the same laser exposure (Fig. 4b). It indicates the generating heat mainly results from the light-harvesting of THPG vesicles and subsequent conversion to thermal energy, but not from the laser itself. An average thermal equilibration time constant (*τ*) related to radiative heat transfer was calculated to be 80.3 s according to the fitting curve in Fig. 4c. Based on this value, the photothermal conversion efficiency of THPG vesicles was calculated to be 44.1% by combining the Eqs. 1–4 (shown in the methods)^16,35–37^. It is higher than most of the organic photothermal agents reported in literatures^4^. THPG vesicles also show excellent photothermal stability. The equilibrium temperature of THPG vesicles remained about 46.1 °C after three heating and cooling cycles under 635 nm laser (200 mW cm^−2^), and there was only a negligible drop compared with the initial temperature of 46.8 °C (Fig. 4d).

To our knowledge, THPG vesicles represent the first polymer vesicles with such excellent photothermal properties, which should be owing to their unique self-assembled structures. The good membrane fluidity of hyperbranched polymer vesicles facilitates the supramolecular polymerization of porphyrin moieties inside the vesicles into ordered nanofilament structure, which greatly enhances the photothermal property of vesicles. As a control experiment, spherical micelles were prepared by using a fast precipitation method from the same THPG polymers, as shown in the TEM image (Supplementary Fig. 13a). Under the same laser irradiation, the equilibrium temperature of 0.3 mg mL^−1^ THPG micelles solution only reached 42.5 °C (Fig. 4b), which is lower than that of 0.3 mg mL^−1^ THPG vesicle solution. Besides, the relatively lower fluorescence self-quenching ratio of THPG micelles ca. 720 than that of THPG vesicles (1100) suggests the less ordered π–π stacking of porphyrin units in the micelles (Supplementary Fig. 13b). These results demonstrated that the vesicular structure and the ordered nanofilaments inside vesicle membranes formed by supramolecular polymerization of porphyrin units both contribute to the outstanding photothermal property of THPG vesicles. We also used the 1,3-diphenylisobenzofuran (DPBF) as the photosensitizer to evaluate the photodynamic effect of THPG vesicles by monitoring the generated singlet oxygen (^1^O_2_) under the laser irradiation at 635 nm (200 mW cm^−2^)^38,39^. The photodynamic ability of THPG vesicles was significantly inhibited due to the strong π–π stacking between porphyrins in the vesicles (Supplementary Fig. 14a), and it was significantly recovered (Supplementary Fig. 14b) when the THPG vesicles were disassembled into polymers.

The stability of nanovesicles in a physiological environment with albumin was evaluated by the absorption intensity ratio between J-aggregates (463 nm) of THPG vesicles and the THPG unimers (425 nm) throughout 48 h (*n* = 3, Supplementary Fig. 15)^24,25^. As a result, the ratio shows only slight change both in PBS and PBS with 10% fetal bovine serum (FBS), which suggests the high optical stability of THPG vesicles in a physiological environment. Such superior stability should be attributed to several aspects, including the strong π–π stacking interaction between porphyrins in ordered nanofilaments inside vesicle membranes, multi-layer membrane structure, and hydrophobic interaction between hydrophobic units.

### In vitro cell experiments

The potential cytotoxicity of THPG vesicles in vitro was assessed by methyl thiazolyl tetrazolium (MTT) assay against NIH/3T3 mouse embryonic fibroblast cells. As depicted in Supplementary Fig. 16, the relative viabilities of 3T3 cells after 48 h incubation with THPG vesicles remained about 86.0% even at a high concentration of THPG vesicles up to 0.2 mg mL^−1^. It suggests that THPG vesicles have appreciable non-toxicity and excellent biocompatibility for NIH/3T3 under our tested concentrations, thus allowing its further biological applications such as PTT here.

**Fig. 5:**
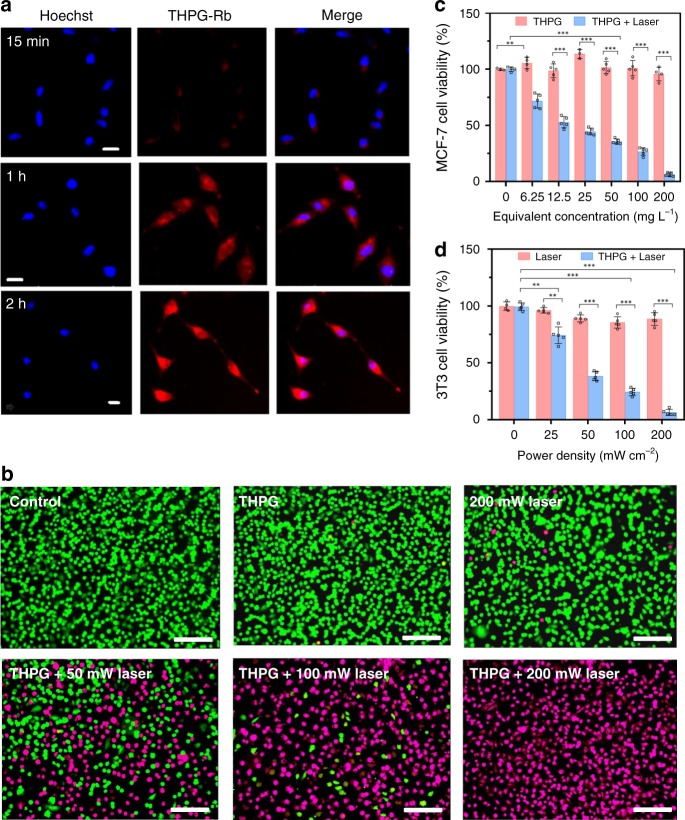
In vitro cell evaluation of biosafety and photothermal effect of the THPG vesicles. **a** The representative CLSM images of cell uptake of THPG-Rb vesicles at various time points by MCF-7 cancer cells. The magenta pink color (recoloring from red color) originates from THPG-Rb vesicles and the blue color is from Hoechst in cell nuclei. The scale bar is 20 μm. Similar images were obtained for more than two times experiments. **b** The representative CLSM images of MFC-7 cells co-stained by calcein AM and propidium iodide (PI) after various experiment conditions indicated. Green and red colors represented live and dead cells, respectively (Scale bar: 150 μm). Similar images were obtained for more than two times experiments. **c** Relative viabilities of MCF-7 cells incubated with various THPG amount with or without 635 nm laser irradiation at 200 mW cm^−2^ for 10 min (*n* = 5 biologically independent cells). **d** Relative viabilities of MCF-7 cells with various laser irradiation power with or without THPG vesicle treatments (*n* = 5 biologically independent cells). Data are presented as mean values +/− S.D., and *P* values are calculated by two-tailed Student’s *t*-test **P < 0.005; ***P < 0.001.

To elucidate the internalization of THPG vesicles by the cancer cells, the MCF-7 cancer cells were incubated with Rb-loaded THPG (THPG-Rb) vesicles at 37 °C and then monitored using confocal laser scanning microscopy (CLSM) over 15 min, 30 min, 1 h, and 2 h. As shown in Fig. 5a, the blue fluorescence was ascribed to cell nuclei counterstained with Hoechst 334221, and the continued increase of red fluorescence from THPG-Rb was observed in the cells with increasing the incubation time from 15 min to 2 h. It indicated the THPG-Rb vesicles were effectively uptaken by MCF-7 cells within 2 h. Consistent with the fluorescence imaging results, the flow cytometry analysis assay exhibited effective cellular uptake of THPG vesicles by MCF-7 cells within 2 h (Supplementary Fig. 17a). Interestingly, the uptake of THPG vesicles into MCF-7 cells was greatly enhanced under the light laser irradiation (Supplementary Figs. 17b, 18), which might result from the promoted membrane mobility of cells by laser for increased endocytosis.

We also performed several control experiments to study the cell internalization process of THPG-Rb vesicles. The flow cytometry analysis of MCF-7 cells incubated by free Rb (Supplementary Fig. 19) shows the uptake of THPG-Rb vesicles is much easier than that of free Rb. The 3D CLSM image indicated the internalized vesicles were mainly located in the cytoplasm rather than the nuclei of the MCF-7 cell after 2 h incubation with THPG-Rb vesicles (Supplementary Fig. 20a, b), while the free Rb preferred to the nuclei than to the cytoplasm for the MCF-7 cells incubated with free Rb (Supplementary Fig. 20c, d)^40^. Furthermore, THPG vesicles w/o Rb internalized by the MCF-7 cancer cells are very stable within 4 h inside cells to keep the fluorescence quenched according to the CLSM measurements (Supplementary Fig. 21) and flow cytometry assays (Supplementary Fig. 22). All these control experiments from Supplementary Figs. 19, 20 indicate the facile internalization of THPG vesicles into the MCF-7 cells through incubation, and the red fluorescence observed from Fig. 5a and Supplementary Fig. 18 should be attributed to the Rb loaded in THPG vesicles but not from THPG vesicles themselves or the free Rb released from vesicles. However, when THPG vesicles were incubated with MCF-7 cells for 24 h, a certain degree of red fluorescence was observed in the cells according to both the CLSM image (Supplementary Fig. 21) and the flow cytometry analysis assay (Supplementary Fig. 22). It might result from the partial disassembly of THPG vesicles into unimers, and thus the red fluorescent signal was partially recovered.

The calcein-AM/propidium iodide (PI) double staining^41^ MCF-7 cell experiments were carried out to allow the direct visualization of the PTT performance of THPG vesicles in vitro. It has been well accepted that AM with green fluorescence stains live cells and PI with red fluorescence (recoloring to be magenta pink color here) stains dead cells. As seen in Fig. 5b, the MCF-7 cells were very healthy with no magenta pink fluorescence in the control experiment. Besides, it was found that neither the THPG vesicles nor the 635 nm laser irradiation alone could induce prominent cell necrosis. However, for the MCF-7 cells incubated with THPG vesicles at a concentration of 200 mg mL^−1^ and irradiated with the laser for 10 min together, the number of dead cells increased with the increase of laser power. When the laser irradiation power reached 200 mW cm^−2^, no green fluorescence was observed in the cells, revealing all MCF-7 cancer cells were killed.

Moreover, we employed the MTT assay further to quantify the PTT performance of THPG vesicles in vitro. MCF-7 cells were incubated with THPG vesicles at the concentrations varying from 0 to 200 mg mL^−1^ with or without 635 nm laser irradiation at 200 mW cm^−2^ for 10 min. About 93.7% MCF-7 cells were thermally ablated under the optimal condition of 200 mg mL^−1^ THPG vesicles with laser power at 200 mW cm^−2^ for 10 min. In contrast, the MCF-7 cells with 200 mg mL^−1^ THPG vesicle incubation without laser irradiation only induced 3.1% death (Fig. 5c). Besides, more MCF-7 cells incubated with THPG vesicles were killed with the increase of the illuminated laser power, while the MCF-7 cells without THPG vesicles incubation survived for 86.5% even under the 200 mW cm^−2^ laser illumination (Fig. 5d). These quantitative cell viability results agree well with the AM/PI double staining assay: the THPG vesicles internalized by the MCF-7 cells can absorb light energy from the laser and then convert it into heat effectively, which triggers the death of the cancer cells. Based on the above results, we can conclude that as-prepared THPG vesicles present excellent PTT ablation efficacy in vitro.

### In vivo animal experiments

**Fig. 6:**
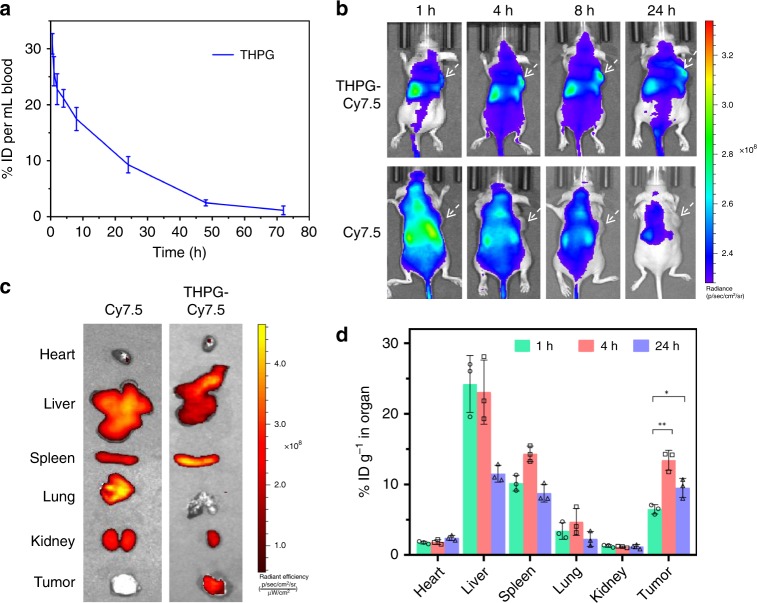
The in vivo pharmacokinetic, imaging, and biodistribution studies of the THPG vesicles. **a** Blood clearance curve as a function of time after tail intravenous injection of THPG vesicles in rat (*n* = 3 biologically independent rat) (ID stands for injected dosage). **b** In vivo fluorescence images of MCF-7 tumor bearing mice with different times after intravenous injection of THPG-Cy7.5 vesicles and Cy 7.5, respectively (Ex: 745 nm, Em: 820 nm; mode: fluorescence). White arrows were used to indicate tumors location. **c** Ex vivo images of isolated organs and tumors at 24 h post-injection of THPG-Cy7.5 vesicles and Cy 7.5, respectively. **d** Biodistribution of THPGs at 1, 4, and 24 h post-injection. (*n* = 3 biologically independent mice). Data are presented as mean values +/− S.D., and *P* values are calculated by two-tailed Student’s *t*-test *P < 0.05, **P < 0.005.

Pharmacokinetic studies were performed to analyze the blood retention of THPG vesicles. For the experiments, SD rats (~200 g) were randomly divided into two groups (*n* = 3). The rats in the experiment group were treated with THPG vesicles in PBS solutions via tail intravenous (i.v.) injection at a dose of 8 mg kg^−1 42^, and the rats without any treatment were regarded as a control group. The blood circulation curve was shown in Fig. 6a and the blood circulation half-life (*t*_1/2_) of THPG vesicles was calculated to be 29.5 h^43^, which is relatively higher than that of most self-assembled organic nanoparticles^16,25,44^. It suggests that THPG vesicles have prolonged blood circulation, probably due to the excellent structural stability even in the presence of albumin in the physiological environment (Supplementary Fig. 15). Besides, the concentration of THPGs in the blood at the post-injection of 72 h was much lower than that at the initial period, indicating that THPG vesicles can be gradually cleared away from the rat body.

Animal fluorescence imaging was performed by the intravenous injection of THPG-Cy7.5 vesicles, the THPG vesicles encapsulated with a near-infrared dye of Cy7.5, into MCF-7 tumor-bearing mice (Fig. 6b). After post-injection of 24 h, we found that the fluorescence signal of free Cy7.5 around the body was much weaker than that of THPG-Cy7.5 vesicles, suggesting the longer retention of THPG-Cy7.5 vesicles in vivo and the rapid clearance of free Cy 7.5 due to the small molecule nature. Moreover, almost no accumulation of free Cy7.5 in the tumor tissue was found within 24 h post-injection. In contrast, the fluorescence signals of THPG-Cy7.5 vesicles increased from 1 to 8 h in the tumor location and were still remarkable after 24 h. Furthermore, the ex vivo organ images of the heart, liver, spleen, lung, and kidney and tumor after 24 h post-injection of THPG-Cy7.5 vesicles were also recorded (Fig. 6c), and the results showed that the fluorescence signals in liver, spleen and tumor were remarkable even after 24 h. On the contrary, there was no fluorescence in the tumor from the free Cy.7.5 group.

The accumulated amount of THPG polymers in the tumors and other major organs was further studied quantitatively. For the experiments, the mice bearing MCF-7 tumors were injected intravenously with THPG PBS solutions, and then mice were sacrificed (three per time point) at post-injection time points of 1, 4, and 24 h. The bio-distribution trends of THPG polymers in the organs (Fig. 6d) are generally consistent with those of the FL imaging experiments (Fig. 6b, c). After 1 h post-injection, THPGs accumulated in the liver, followed by spleen, tumor, lung, heart, and kidney. The amount of THPGs in tumors reached a maximum of 13.5% ID g^−1^ (ID stands for injected dosage) after 4 h post-injection. Subsequently, the THPG contents in most of the organs notably decreased after 24 h. However, the decreasing degree in the tumor was smaller than that in the liver and spleen.

All these bio-imaging and bio-distribution experiments suggest the gradual accumulation of THPG vesicles at the tumor tissue within 24 h, probably resulting from the enhanced permeability and retention (EPR) effect^45,46^ and the long blood residence of THPG vesicles. In addition, these experiments also provided useful information to optimize laser irradiation time points on local tumor tissue and to maximize PTT therapeutic efficacy as well as minimize damage to surrounding healthy tissue.

**Fig. 7:**
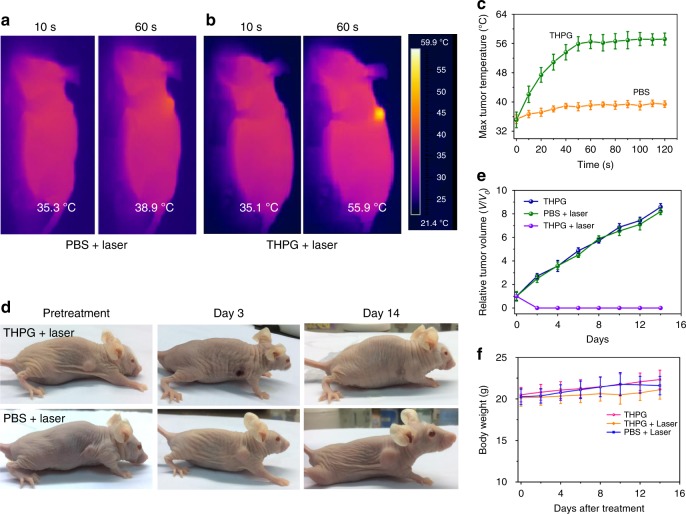
In vivo evaluation of photothermal effect of THPG vesicle. Thermal images of MCF-7 tumor-bearing mice after injection with PBS (**a**) and THPG solutions (**b**) and irradiated under 635 nm laser at 200 mW cm^−2^ for 10 min. **c** Quantitative analysis of temperature changes in tumor area during laser irradiation monitored by the thermal camera (*n* = 5 biologically independent experiments. **d** Representative photographs of mice after PTT treatments. **e** Tumor growth inhibition profiles of different groups of tumor-bearing MCF-7 mice after treatments. (*n* = 5 biologically independent mice). **f** Body weight curves of MCF-7 tumor-bearing mice upon different treatments (*n* = 3 biologically independent mice). Data are presented as mean values +/− S.D.

Subsequently, we further investigated the PTT efficacy of THPG vesicles in vivo. MCF-7 tumor-bearing mice were randomly divided into two groups: mice in one group were injected intravenously with THPG vesicles (200 μL of a 2 mg mL^−1^ solution for each mouse), mice in the other group were injected with saline as control. After the post-injection of 4 h, the tumor tissues of these mice were exposed to 635 nm lasers at the power density of 200 mW cm^−2^, and the temperature alteration at the tumor sites was monitored in real-time by an IR thermographic camera. As shown in Fig. 7a, only a slight temperature rise from 35.3 to 38.9 °C was observed within 60 s’ laser irradiation at the tumors of mice in the control group. However, in the same conditions, the mice injected with THPG vesicles exhibited a significant temperature increase from 35.1 to 55.9 °C (Fig. 7b). The real-time temperature rise profiles of the tumors were also shown in Fig. 7c, which further indicated the high photo-heat transfer efficiency of THPG vesicles.

We also recorded the tumor evolution of mice over 14 days by using an optical camera (Fig. 7d). Herein, in the treatment group, laser irradiation was conducted for 10 min in tumors after the 4 h post-injection of THPG vesicles into mice, and no follow-up treatment was performed. It was found that the tumors of mice injected with PBS continued to increase. In marked contrast, the tumors in the treatment group formed eschars after two days, and the eschars were fallen off naturally after 14 days without recurrence. The tumor volume was calculated according to the following equation: (tumor width)^2^ × (tumor length)/2. Relative tumor volume was calculated by the equation of *V*/*V*_0_ (*V*_0_ was the tumor volume when the treatment initiated)^11^. After 14 days, the volume of tumors in control groups increased to several folds. However, the size became zero in the treatment group after two days (Fig. 7e). Besides, the status of the mice was carefully monitored after treatment, and neither notably reduced body weight of mice nor apparent abnormal behaviors of mice were observed within 14 days (Fig. 7f), suggesting the THPG vesicles possess negligible biotoxicity and acceptable biocompatibility.

**Fig. 8:**
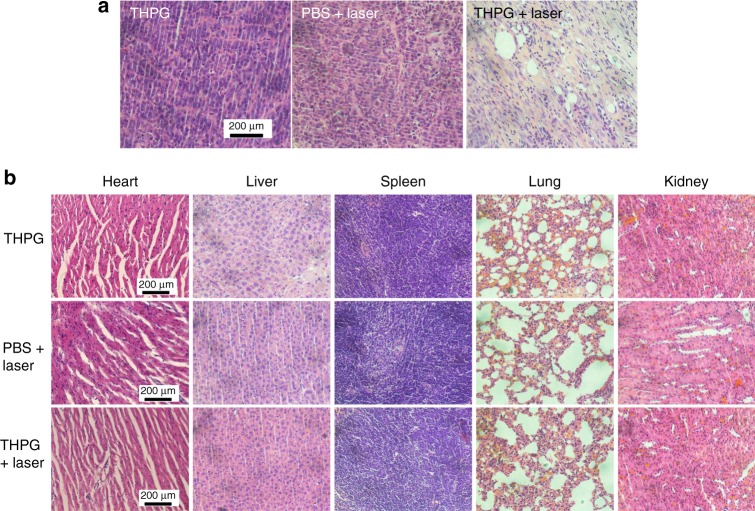
Histology analysis of therapeutic effects and in vivo safety of THPG vesicles. **a** Representative H&E stained images of tumor sections collected from different groups of mice at 2 days after laser irradiation (*n* = 3 biologically independent mice). **b** Representative H&E stained images of major organs (heart, liver, spleen, lung, and kidney) harvested from mice in control group (THPG only and PBS+laser) and treatment group (THPG+Laser) at 14 days after treatment (*n* = 3 biologically independent mice).

Tumor tissues at two days after post-treatment were gathered and determined by hematoxylin and eosin (H&E) staining assays^47^ to further study the PTT effects at the histological level. As expected, the tumor tissues from the treatment group (THPG+laser) show evident damages when compared with those of two other control groups (THPG, PBS+ laser), such as the widening of intercellular space, cellular shrinkage and destruction of the tumor extracellular matrix (Fig. 8a). Furthermore, the principal organs of mice (heart, liver, spleen, lung, and kidney) at treatment and control groups were also examined by H&E staining after 14 days. As shown in Fig. 8b, no detectable damages or inflammatory lesions in the organ tissues from mice were observed in THPG+laser group. There were no remarkable differences between the organ tissues of the treatment group and control groups. It suggests that the THPG vesicles administrated into mice have negligible biological toxicity to mice at a given dose, and the photo-generated heat is localized in tumor tissues to prevent excessive off-target damage.

## Discussion

In summary, herein, we report a “supramolecular polymerization-enhanced self-assembly” process to construct hyperbranched polyporphyrin vesicles for highly efficient photothermal therapy. The as-prepared polymer vesicles with an average diameter of 176 nm possess excellent biocompatibility and structural stability. Most importantly, due to the inherent good membrane fluidity of hyperbranched polymer vesicles, the porphyrin units in the vesicle membranes can further self-assemble into nanofilament-like supramolecular polymers driven by the 1D directional π–π stacking interactions, which leads to a very high fluorescence self-quenching ratio up to ca. 1100 and photothermal conversion efficiency of 44.1% in vitro. Besides, the hollow structure endows the vesicles with fluorescence monitoring and imaging-guidance capacity through the encapsulation of hydrophilic functional molecules such as Rhodamine b and Cy 7.5. As a result, the as-prepared polymer vesicles have shown excellent thermal ablation efficacy for both tumor cells and tumors in mice, thus making them very potential in photothermal therapy. The supramolecular polymerization-enhanced self-assembly process, as reported here, has combined the covalent polymer self-assembly and noncovalent supramolecular polymerization, and such a combination can strengthen the final self-assembled structure and enhance the functions in a synergistic way, which might be a promising way to extend the applications of the obtained supramolecular materials.

## Methods

### Synthesis of hyperbranched multi-porphyrin polymers (THPGs)

Most of the chemicals were obtained from Sigma and used without further purification. KH in mineral oil was purchased from Acros Company and washed by distilled tetrahydrofuran (THF) for five times. Dichloromethane (CH_2_Cl_2_), dimethylformamide (DMF), and THF were purified with CaH_2_, and THF was further distilled with sodium wire before use. Other chemicals were purchased from Sinopharm Chemical Reagent Company (SCRC) and used as received.

### Synthesis of 5, 10, 15, 20-Tetrakis (4-hydroxyphenyl) zinc porphyrin (ZnTHPP)

ZnTHPP was synthesized according to the following modified method^31^. THPP (50 mg) was reacted with zinc acetate (Zn(CH_3_COO)_2_, 1.45 g) in CH_2_Cl_2_ (50 mL) and DMF (50 mL) solvent at 60 °C overnight under vigorously stirring with the nitrogen atmosphere protection. The solution was precipitated in pure H_2_O, filtered and washed with H_2_O for five times and then vacuum dried to obtain purple solid.

### Synthesis of THPG polymers

Anion-initiated polymerization by di-epoxy ether as A_2_ monomer and ZnTHPP as B_4_ monomer was conducted as follows to prepare an amphiphilic hyperbranched multi-porphyrin polymer (Supplementary Fig. 1). In a glove box, the solution of ZnTHPP (0.2 g) in dry distilled DMF (5 mL) was added to a 50 mL one-neck flask with potassium hydride (0.016 g) under vigorous stirring conditions for 1 h at 60 °C, named as A solution. Di-epoxy ether (0.016 g) was dissolved into 10 mL DMF, and then the solution was heated up to 80 °C, following slowly adding A solution into it for 24 h. The feed ratio between di-epoxy ether and ZnTHPP monomers is 2:1. After another 24 h reaction, the DMF was rotary evaporated and the resulted product was dissolved into methanol and dialyzed against (MWCO: 3500 Da) in ethanol for three days, and then freeze-dried in a vacuum.

### Self-assembly of THPGs

The co-solvent method of self-assembly was used to prepare THPG vesicle in this work. Firstly, amphiphilic THPG (10 mg) was dissolved in good solvent DMSO (5 mL), and the poor solvent deionized water (10 mL) was slowly added into the solution at a constant speed during 12 h under strongly magnetic stirring. The mixture solution was dialyzed against distilled water for 48 h. The disassembly experiments were conducted by adding 5% Triton X-100 as detergent solutions.

### Computer simulation of THPGs

The atomistic simulation was carried out with the General Amber force field (GAFF)^48^ by using GROMACS package (Version 4.5.4)^49^. The simulation was performed for 100 ns with 1 fs time step under NPT ensemble at 303 K, and Berendsen barostat^50^ and V-rescale thermostat were used to control the system pressure and temperature, respectively. The cutoff radius of 12 Å was used for the vdW interaction calculations, and the electrostatic interactions were evaluated by particle mesh Ewald (PME) method with a direct space cutoff distance of 12 Å. Long-range dispersion corrections were applied to both energy and pressure calculations.

### Photothermal efficiency calculation of THPG vesicles

0.2 mL solution containing PBS and 1 mg mL^−1^ THPG micelle and THPG vesicles were dropped in impending 1 mL transparent plastic centrifugal tube, respectively. Then the solutions were exposed to 635 nm light at the power density of 200 mW cm^−2^ for 5 min and simultaneously imaged by an IR thermal camera (FLIR, USA). To test the photothermal stability of THPG vesicles, three cycles of heating and cooling were recorded.

The photothermal conversion efficiency (*η*), was calculated using the following Eq. (1)^16,33–35^.

1 $$\eta = \frac{{h{\mathrm{A}}\left( {T_{{\mathrm{max}}} - T_{{\mathrm{sur}}}} \right) - Q_{{\mathrm{in}}}}}{{I\left( {1 - {\mathrm{10}}^{{\mathrm{ - A635}}}} \right)}},$$

where *T*_max_ is the maximum system temperature, *T*_sur_ is the surrounding environmental temperature, and (*T*_max_ − *T*_sur_) was 22.7 °C. I is the input laser power (in units of mW, 200 mW cm^−2^ in a spot size of 6 mm diameter) and A_635_ is the absorbance of THPG vesicle solution at the excitation wavelength of 635 nm, which is 0.62 determined by UV–Vis experiment. *Q*_in_ (in units of mW) is the heat input from the incident laser light by the solvent and container, and it is 5.4 × 10^−4^ I J s^−1^determined by using a quartz cuvette cell containing pure water without the vesicles. *h* is the heat transfer coefficient, *A* is the surface area of the container, and the value of *h*A can be obtained from Fig. 3c and Eq. 2.

2 $$h{\mathrm{A = }}\frac{{m_{\mathrm{D}}C_{\mathrm{D}}}}{{\tau _{\mathrm{s}}}}$$

where *m*_D_ and *C*_D_ are the mass (0.2 mg) and heat capacity (4.2 J/g °C) of the solvent of deionized water, respectively. *τ*_s_ is the time constant of the sample system. It can be calculated by Eq. (3)

3 $${\mathrm{t}} = - \tau _{\mathrm{s}}\ln \left( \theta \right)$$

Here, a dimensionless parameter *θ* was introduced and expressed as Eq. (4):

4 $$\theta = \frac{{T - T_{{\mathrm{sur}}}}}{{T_{{\mathrm{max}}} - T_{{\mathrm{sur}}}}}$$

As a result, the *τ*_s_ can be calculated to be 80.39 s according to Eq. (3) and Fig. 4c. Thus, the photothermal conversion efficiency (*η*) of the THPG vesicles was calculated to be 44.1%.

### Cell culture and MTT assays

The mouse fibroblast NIH/3T3 cell and human breast carcinoma cell lines MCF-7 (Shanghai Institute of Cell Biology, the Chinese Academy of Sciences) were cultured in DMEM medium supplemented with 10% FBS, 5% penicillin/streptomycin (PAA Laboratories) in a humidified atmosphere containing 5% CO_2_ at 37 °C to evaluate the biocompatibility and the photothermal therapy of the THPG.

### In vitro cellular cytotoxicity

NIH/3T3 cells were seeded at a density of 6000 cells per well in 96-well plates for 24 h. After 24 h, the supernatant was removed and fresh culture medium containing serial concentrations of THPG were added into each well. The cells in each well were cultured for 48 h and then treated by 20 μL of 5 mg mL^−1^ MTT in PBS. The cells were incubated for another 4 h and generated blue formazan crystals. The medium was removed carefully to discard unreacted MTT. The blue formazan crystals in each well were dissolved by 200 μL DMSO and the absorbance was detected on a microplate reader (BioTek® SynergyH4 hybrid) at a wavelength of 490 nm.

### Photothermal therapy in vitro

MCF-7 cells were seeded at a density of 6000 cells per well in 96-well plates for 24 h. Then, the supernatant was removed, and THPG with various concentrations (0, 6.25, 12.5, 25, 50, 100, and 200 mg L^−1^) were added into each well. After 24 h of incubation, the cells in photothermal therapy groups were exposed to 635 nm light at the power density of 50 mW cm^−2^ for 5 min. Besides, the cells treated by THPG (50 mg L^−1^) were exposed to 635 nm light at different power densities of 0, 25, 50, 100, 200 mW cm ^−2^ for 5 min. The cells in control groups without light exposure remained in the dark. After being further incubated for another 24 h, the viabilities of cells were measured by the standard MTT assays mentioned above.

### Singlet oxygen detection

The singlet oxygen (^1^O_2_) generation of THPG was evaluated by singlet oxygen capture agent (1,3-diphenlisobenzofuran (DPBF)). Fluorescence spectra of DPBF were recorded in the presence of THPG vesicle in PBS solution and 5% Triton X-100 solution (containing 1% DMF to dissolve DPBF) under irradiation with 635 nm laser light as a function of different irradiation time (*λ*_ex_ = 360 nm, *λ*_em_ = 470 nm).

### Cell uptake

MCF-7 cells were seeded at a density of 3.0 × 10^5^ cells per well in six-well plates and cultured for 24 h. The cells were incubated with Rb loaded THPG (50 mg L^−1^), THPG vesicles (50 mg L^−1^) and pure Rb with similar absorption intensity of Rb with Rb loaded THPG for 15 min, 1 h and 2 h, respectively. To investigate the effect of laser exposure to the cell uptake of Rb loaded THPG nanoparticles, the MCF-7 cells were irradiated by a 635 nm laser at a power density of 50 mW cm^−2^ for 5 min after being incubated with Rb loaded THPG (50 mg L^−1^) for 10 min. The pretreated cells were fixed with 4% paraformaldehyde in PBS for 30 min, washed with PBS for three times, stained with Hoechst 33342 (Sigma-Aldrich) for 5 min, washed with PBS for three times, and then imaged by a confocal fluorescence microscope (LSM510 META, ZEISS). 3D CLSM images of cellular uptake of THPG and Rb were also taken to evaluate their trace of samples in cells.

### Flow cytometry analysis

MCF-7 cells were seeded at a density of 3.0 × 10^5^ cells per well in 6-well plates and cultured for 24 h. The cells were incubated without and with Rb loaded THPG solution (50 mg L^−1^) for 15 min, 30 min, 1 h, 2 h, 4 h, and 24 h, respectively, or free Rb with same concentration for 15 min, 30 min, 1 h, 2 h, 4 h, respectively. To investigate the effect of laser exposure on the cell uptake of THPG-Rb vesicles, the MCF-7 cells were irradiated by a 635 nm laser at a power density of 50 mW cm^−2^ for 5 min after being incubated with Rb loaded THPG solutions (50 mg L^−1^) for 10 min. Then the cells were washed three times with PBS, following trypsinized and re-suspended in 0.5 mL PBS. Data for 1.0 × 10^4^ gated events were gathered (example for the gating strategy of Rb at 2 h was provided in Supplementary Fig. 23. Subsequently, the treated cells were analyzed with a Flow cytometry analysis (BD FACSCalibur, BD Bioscience). The fluorescent intensity was calculated by Cell Quest software and blanked by untreated cells.

### The Calcein-AM/PI costained assay

MCF-7 cells were seeded at a density of 1.5 × 10^5^ cells per well in 12-well plates for 24 h. Subsequently, the cells were incubated with and without Rb loaded THPG (50 mg L^−1^) for 24 h. And then the supernatant was replaced by fresh culture medium. Subsequently, the cells were exposed to 635 nm light at different power densities of 50, 100, and 200 mW cm^−2^ for 5 min. The cells without light exposure were used as control groups. After being further incubated for 4 h, the cells were stained with Calcein-AM/PI for 30 min, washed with PBS for three times, and then imaged by a fluorescence microscope.

### Animal experiments

All procedures of animal experiments were approved by the Shanghai Jiao Tong University Animal Care and Use Committee and all the animals were kept in standardized conditions under Chinese NIH guidelines for the care and use of laboratory research animals. All experiments were performed in accordance with relevant guidelines and regulations. SD rats (~200 g) and female nude mice (5–6 weeks) were supplied by the Chinese Academy of Sciences of Shanghai. The laboratory mice housed in maximum-barrier facilities under 24 °C. 6 × 10^6^ MCF-7 cells suspended in 200 μL PBS were subcutaneously injected into axillary subcutaneous tissue on the frank region of female nude mice (5–6 weeks).

### Pharmacokinetic studies

For pharmacokinetic studies of THPG vesicles, SD rats (~200 g) were randomly divided into two groups (*n* = 3). The rats in the experiment group were intravenous injected (i.v.) with THPG PBS solutions with a dose of 8 mg kg^−1^ via vein, and the rats treated with the same volume of PBS were regarded as the control group. 0.5 mL blood samples were collected from plexus venous in the eyeground and stored in heparinized tubes at 0.5, 1, 2, 4, 8, 24, 48, and 72 h post-injection. Then, 100 μL whole bloods were lysed by 100 μL cold RIPA lysis solution for 15 min in ice boxes, and then 800 μL DMSO was added for extracting THPG polymers. After shaking for 1 min, the supernatant solutions were obtained using centrifugation at 2775 × *g* for 10 min. The samples were then determined using fluorescence spectroscopy (Excitation wavelength = 430 nm). The concentrations of THPG polymers at different time points were calculated from the standard curve.

### Bio-distribution analysis

To assess the tissue distribution of THPG vesicles, the samples in PBS solutions were intravenously injected into the mice bearing MCF-7 tumors. Mice were sacrificed (three per time point) at 1, 4, and 24 h time post-injection. Tumors and organs of the heart, liver, spleen, lung, and kidney were harvested, excised, washed using PBS solution. After making them dry, putting organs (only a small part of livers) and tumors into 2 mL bead-containing homogenizer tubes and the weight of tissues were recorded and then 1 mL DMSO was added into every tube. The tissues were homogenized using a homogenizer for 3 min at a frequency of 65 Hz. The process was repeated three times for each sample. Then the suspensions were centrifuged at 17,500 × *g* at 4 °C for 15 min twice. The supernatant solutions were then determined using fluorescence spectroscopy (*λ*_ex_ = 430 nm). The concentrations of THPG polymers at different time points were calculated from the standard curves previously obtained.

### In vivo photothermal therapy

The mice were randomly assigned to one treatment group with photothermal therapy and two control groups (*n* = 5). Mice bearing MCF-7 tumors were injected with 200 μL of 2 mg mL^−1^ THPG vesicles through the caudal vein. After THPG vesicles accumulated in the tumor site for 4 h, the tumor sites were irradiated with the 635 nm NIR laser at power densities of 200 mW cm^−2^ for 10 min. For one control group, mice were treated with 200 μL saline, were irradiated with the 635 nm NIR laser at power densities of 200 mW cm^−2^ for 10 min at 4 h post-injection. For the second control group, mice were treated with 200 μL of 1 mg mL^−1^ THPG but without laser exposure. The tumor sizes and body weights were measured by a calliper every 2 days for 14 days. The tumor volumes were calculated according to the following equation: (tumor width)^2^ × (tumor length)/2. Relative tumor volumes were calculated as *V*/*V*_0_ (*V*_0_ is the tumor volume when the treatment was initiated)^11^.

### IR thermal imaging

Mice bearing MCF-7 tumors treated with THPG or PBS were irradiated with the 635 nm laser at power densities of 200 mW cm^−2^ for 10 min and simultaneously imaged by an IR thermal camera (FLIR, USA).

### In vivo imaging

Near-infrared fluorescent probe Cy7.5 was loaded into THPG vesicles through physical entrapment to track the path of THPG in mice. After intravenous injection of 200 μL 1 mg mL^−1^ THPG-Cy7.5 vesicles solution, mice bearing MCF-7 tumors at 1, 4, 8, and 24 h post-injection were imaged by an IVIS Spectrum In vivo Imaging System (Perkin Elmer) (*λ*_ex_ = 745 nm, *λ*_em_ = 820 nm). Besides, mice at 24 h time post-injection were sacrificed, and the tumors and organs of were harvested, excised, washed using PBS solution. Then *ex-vivo* organs and tumors imaging were taken on an In vivo Imaging System under the same experimental parameter with the in vivo experiment.

### Histopathological analysis

Hematoxylin and eosin (H&E) were performed following the manufacturer’s instructions to evaluate histological changes of the tumor, livers, kidneys, spleens, lungs, and hearts of mice. The obtained slices of tumor, livers, kidneys, spleens, lungs, and hearts were examined under a digital microscope (Nikon ECLIPSE Ti-U).

### Reporting summary

Further information on research design is available in the Nature Research Reporting Summary linked to this article.

## Supplementary information

Supplementary Information

Reporting Summary

## Data Availability

The authors declare that the main data supporting the findings of this study are available within the article and its Supplementary Information files. Extra data are available from the corresponding author upon request.
