# Supplementary material for: In situ supramolecular polymerization-enhanced self-assembly of polymer vesicles for highly efficient photothermal therapy
Source: Nat Commun. 2020 Apr 7;11:1724. doi: 10.1038/s41467-020-15427-1 (PMC7138818; doi:10.1038/s41467-020-15427-1)
Supplement: Supplementary file 1 — Supplementary Information [file 41467_2020_15427_MOESM1_ESM.pdf]

Supplementary Information

In-situ Supramolecular Polymerization-Enhanced Self-Assembly of Polymer Vesicles for Highly Efficient Photothermal Therapy

Liu *et al.*

1. Supplementary Methods

Nuclear Magnetic Resonance (NMR). ^{1}H NMR spectra was recorded using Bruker AVANCEIII 400 spectrometer with DMSO-d6 as solvents at 293K. Tetramethylsilane (TMS) was used as an internal standard.

Gel Permeation Chromatography (GPC). The molecular weights and molecular weight distribution of the products were carried out on a WATERS GPC (DAWN EOS, Wyatt Technology) at 293K with THF as mobile phase at a flow rate of 1.0 mL/min.

Dynamic Light Scattering (DLS). DLS and Zeta potentials were measured in aqueous solutions using a Malvern Zetasizer Nano ZS 90 (Malvern Instruments, Ltd.) equipped with a 4 mW He-Ne laser light. All samples were measured with a scattering angle of 90º.

Scanning Electron Microscopy (SEM). SEM measurements were performed on NOVA Nano SEM 230 (FEI). One drop of the sample solution was placed onto the surface of cleaned silicon silde and the samples were freeze-dried in vacuum at -40 ºC for 48h. The samples were coated with platinum for 60s before measuring.

High Resolution Transmission Electron Microscopy (HRTEM). HRTEM measurements were performed with a JEOL JEM-2100F instrument at a voltage of 200 kV. The TEM samples were prepared by deposit one drop of solutions onto carbon-coated copper grids, and the grids were freeze-dried in vacuum at -40 ºC for 24h. The EDS line analysis is also conducted in the TEM system to identify the distribution of chemical element in the observed particles.

Ultraviolet-visible (UV-vis) absorption spectra. The UV-vis absorption spectra of sample were measured at 25 ºC in the range of 300–800 nm on a Perkin Elmer Lambda 20 UV-vis spectrometer. The solutions of samples were added to a 1cm quartz cuvette for the measurements.

Fluorescence spectra (FL). The fluorescence spectra were recorded on were carried out on a PTIQM/TM/IM steady-state & time-resolved fluorescence spectro-fluorometer (USA/CAN Photon Technology International Int.). The excitation wavelength was 425 nm.

2. Supplementary Figures

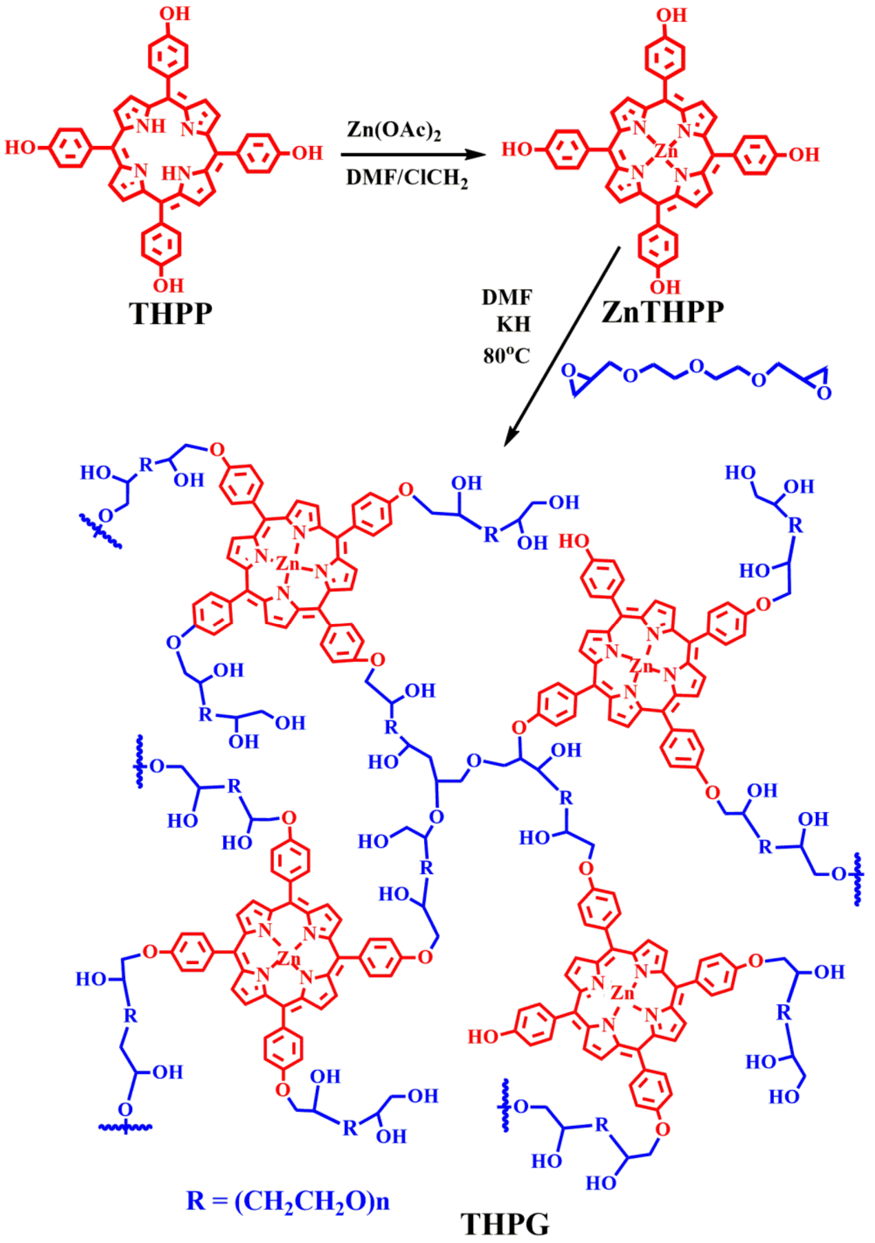

Supplementary Figure 1. Synthetic roadmap of hyperbranched multi-porphyrin polymer THPGs

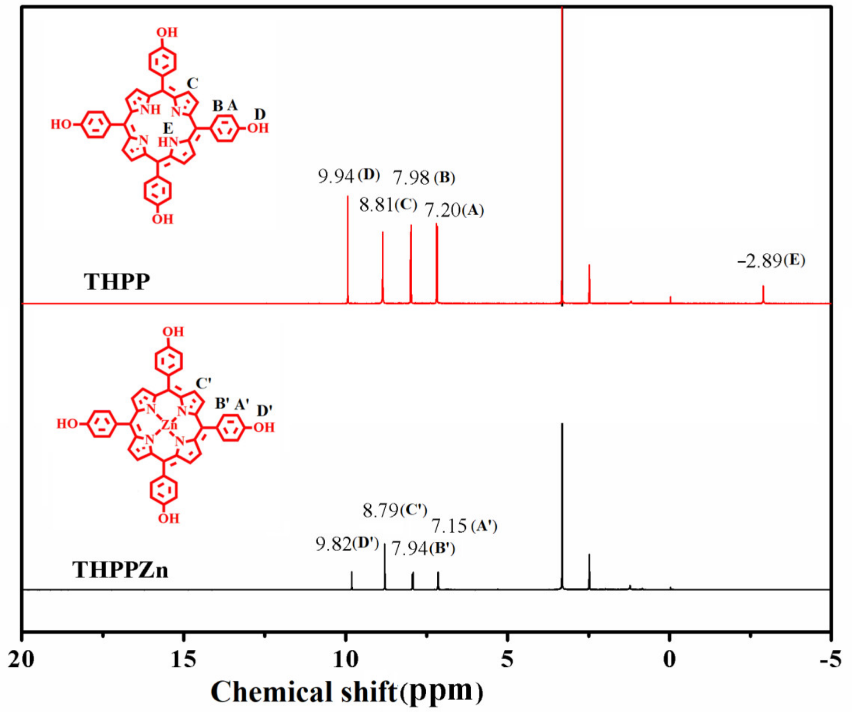

Supplementary Figure 2. The H[1] NMR spectra of THPP and ZnTHPP. The ^{1}H NMR spectrum of THPP had a single peak at -2.89 ppm ascribed to the proton of the free NH_2 in THPP and it disappeared in spectrum of ZnTHPP, which suggested the success of metal coordination reaction. The peak of phenolic hydroxyl in THPP was shifted from 9.94 ppm to 9.82 ppm in ZnTHPP due to the Zn coordination.

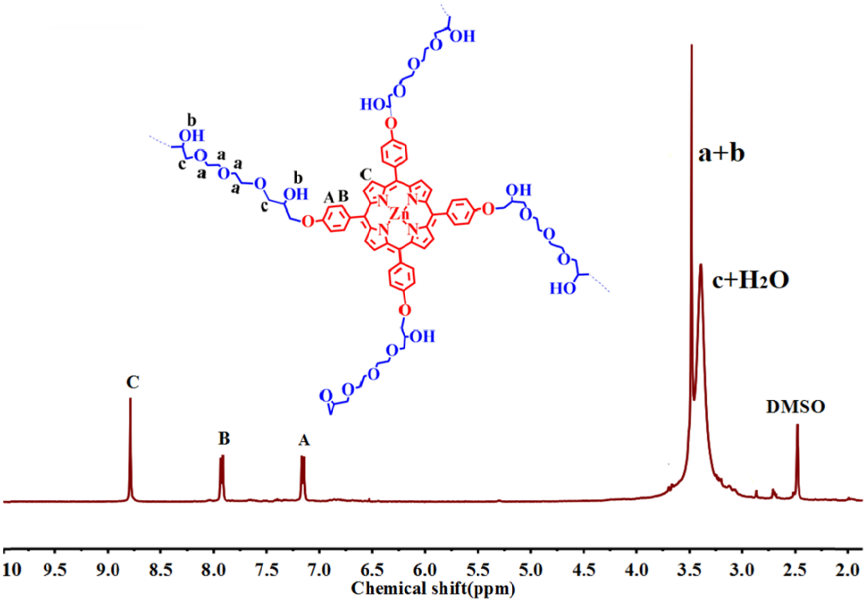

Supplementary Figure 3. The ^{1}H NMR spectrum of THPGs with the feed ratio of ZnTHPP to di-epoxy ether of 1: 2.

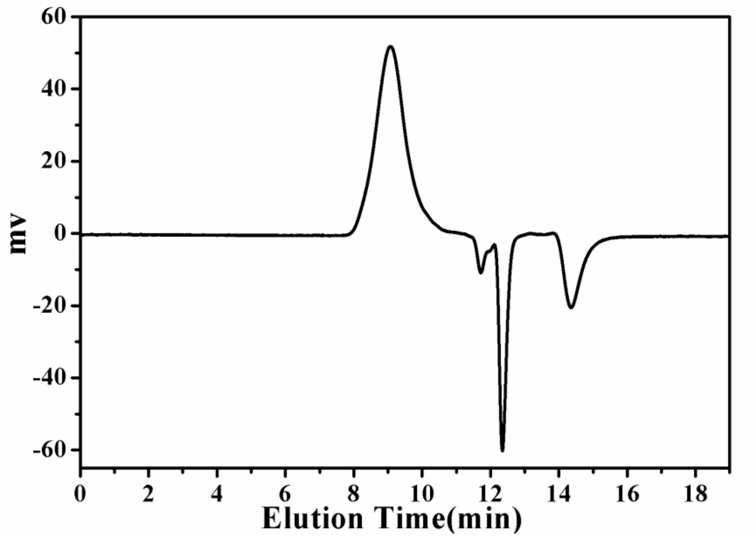

Supplementary Figure 4. The GPC trace of THPGs (THF as an eluting agent)

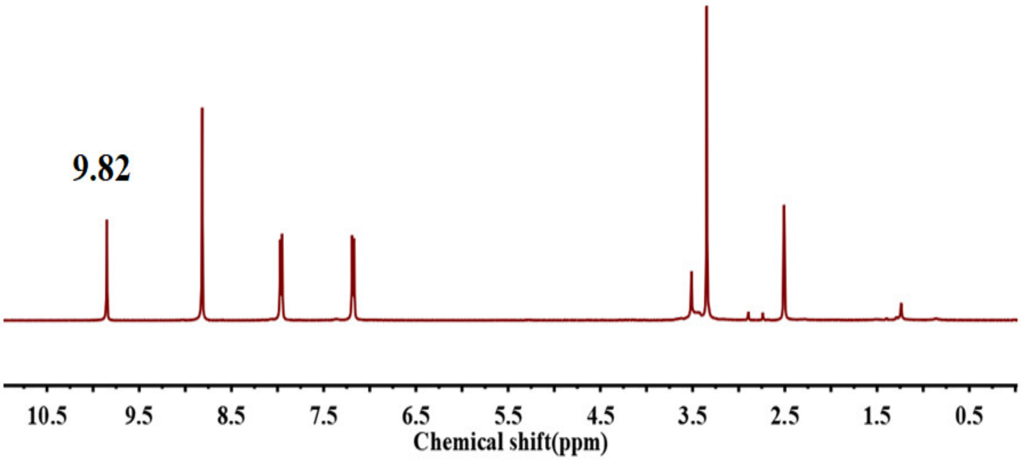

Supplementary Figure 5. The H^1 NMR spectrum of $THPG_{11}$ with the feeding ratio of di-epoxy ether to ZnTHPP of 1: 1 (DMSO-d6, 400 MHz, 298 K). The appearance of the peak at 9.82 ppm indicated the existence of unreacted phenolic hydroxyl groups.

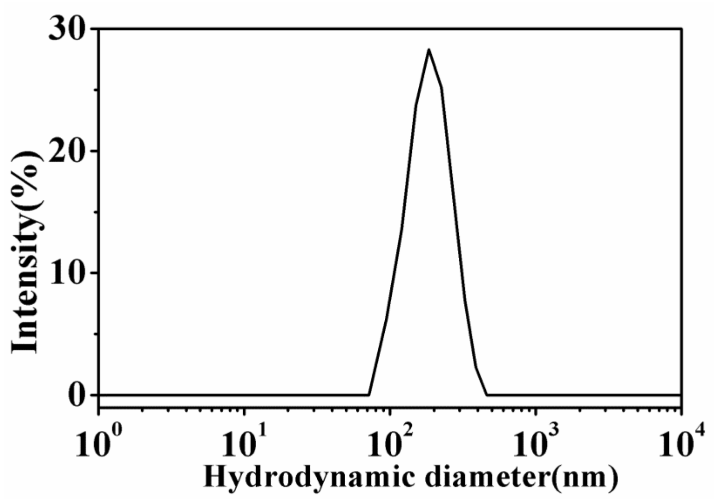

Supplementary Figure 6. The DLS curve of THPG vesicles.

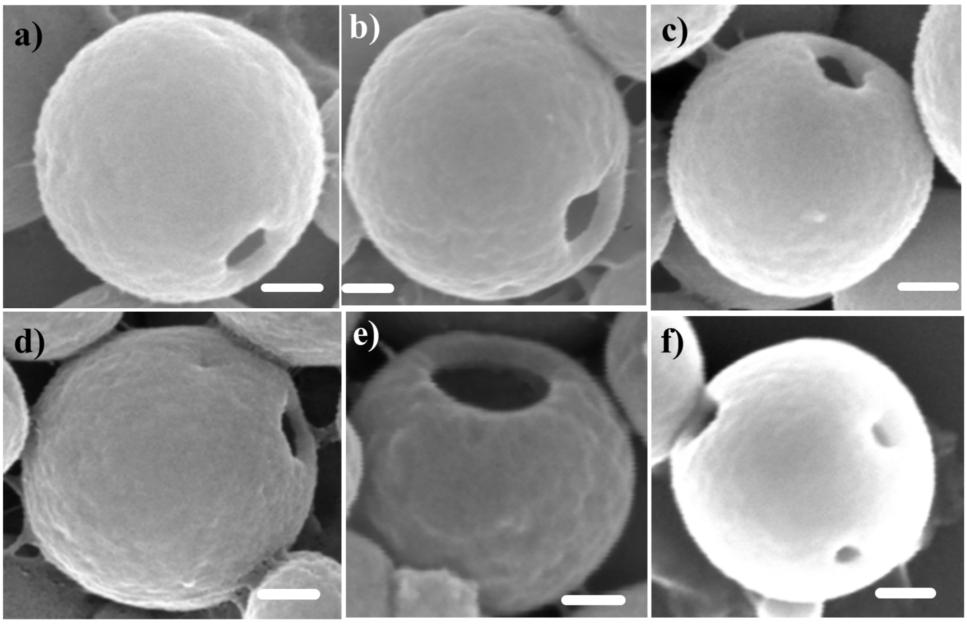

Supplementary Figure 7. Representative magnified SEM images of broken THPG vesicles. The scale bar is 30 nm. Similar SEM images of THPG vesicles were obtained for more than 5 time experiments.

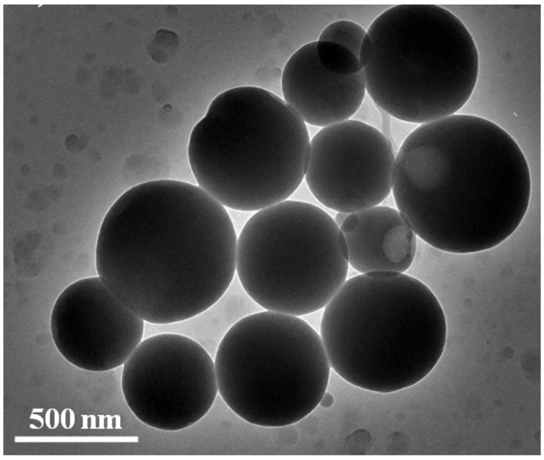

Supplementary Figure 8. The representative TEM image of vesicular self-assemblies from $THPG_{11}$. Similar TEM images of $THPG_{11}$ were obtained for more than 2 time experiments.

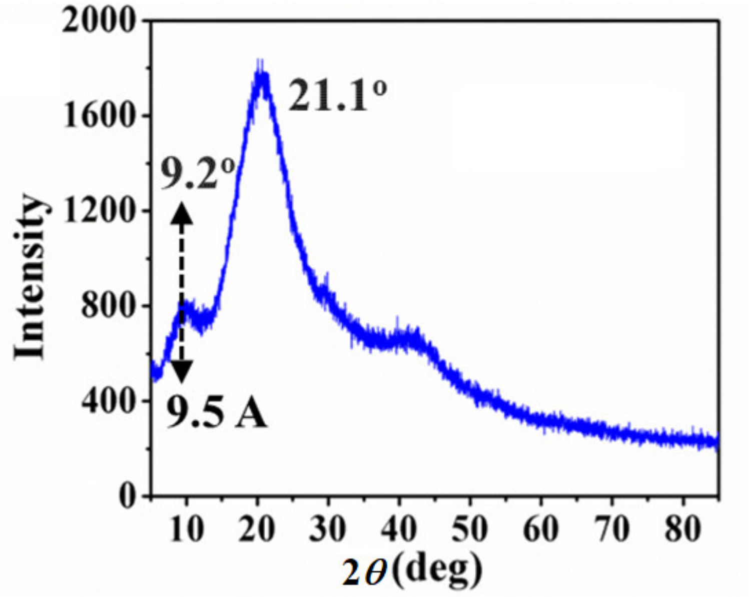

Supplementary Figure 9. The PXRD spectrum of dried THPG vesicles. The first peaks around 9.2° attributed to the thickness of porphyrin nanofilaments, while the peaks around 21.1° attributed to the π-π stacking of porphyrin units.

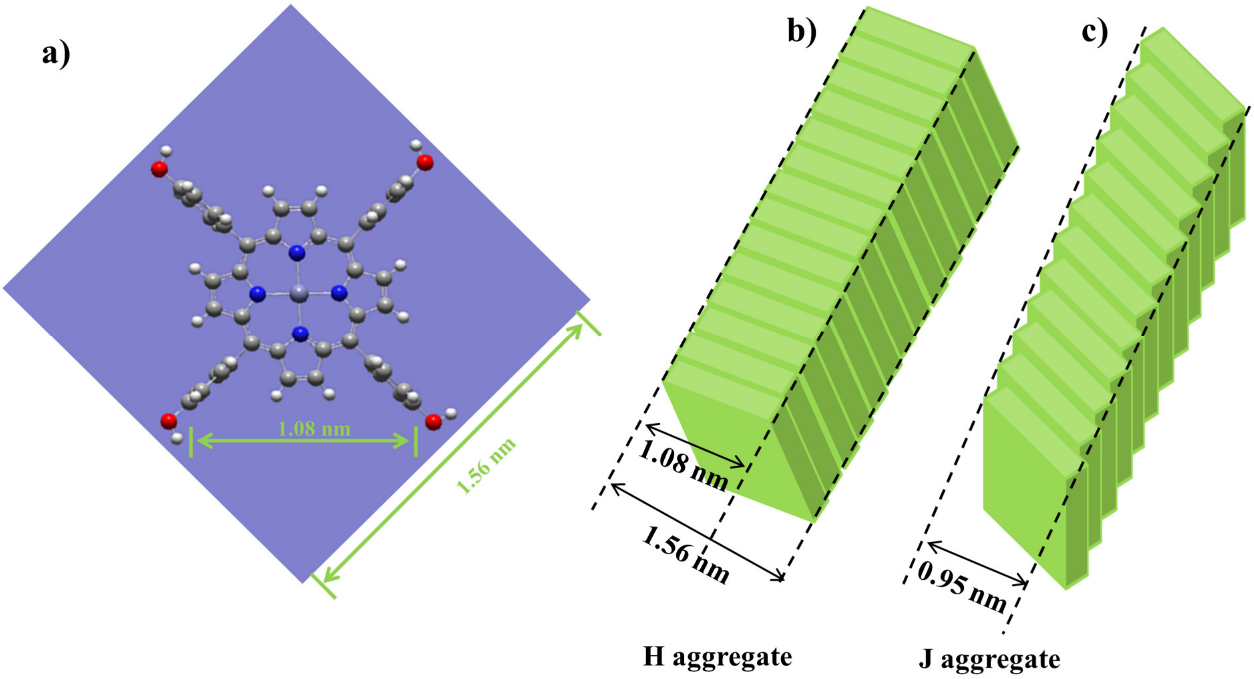

Supplementary Figure 10. (a) The vertical and parallel distances of ZnTHPP molecules. The diagrammatic drawing of H aggregate (b) and J aggregate (c) of ZnTCPP.

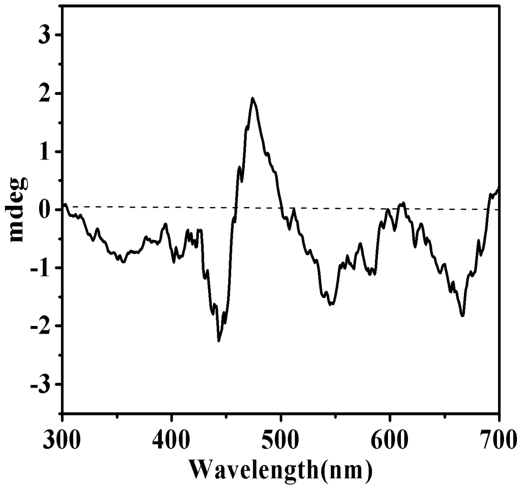

Supplementary Figure 11. The CD spectrum of THPG vesicles as solid powders.

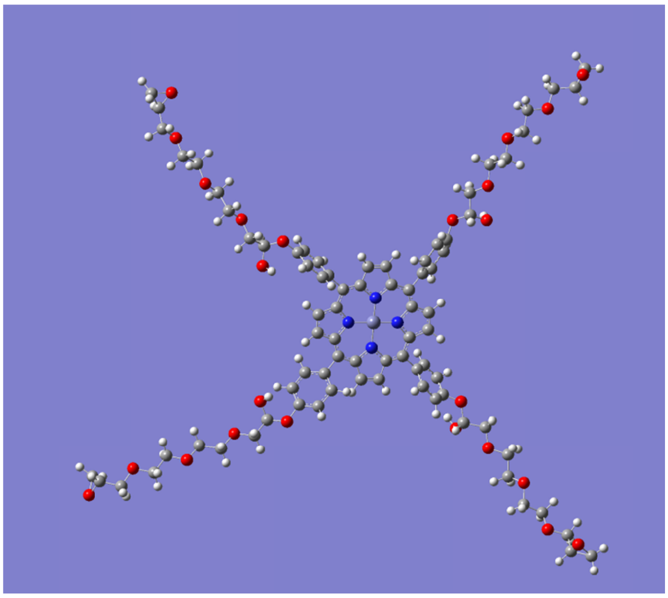

Supplementary Figure 12. The whole atomic formula of the simplified THPG molecules (in which each porphyrin molecule linked four arms). For calculating the π–π interaction, a quantum simulation was carried out with density functional theory by using Gaussian09 (Version D.01)[1] package. The density functional was B97D and the basis set was 6-31G(d) for all elements[2].

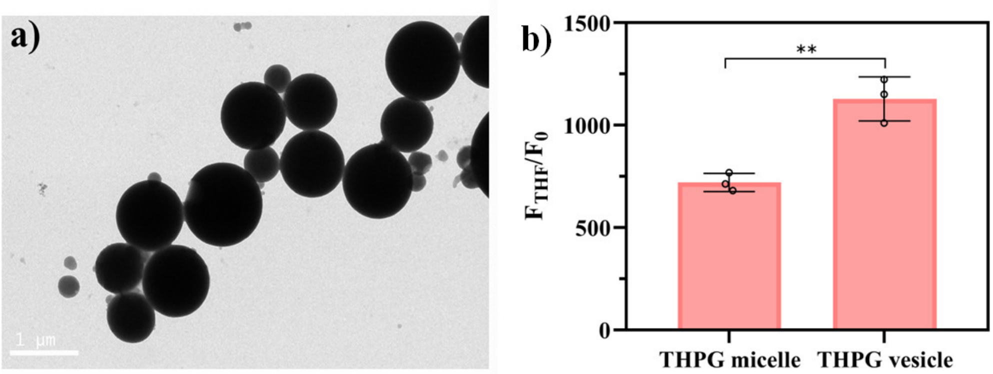

Supplementary Figure 13. (a) The representative TEM image of THPG micelles prepared by a fast precipitation method. Similar TEM images of THPG micelle were obtained for more than 3 times experiments. (b) The fluorescence self-quenching ratios of THPG micelles and vesicles. Data are presented as mean values +/- S.D. from three experiments, and P values are calculated by two-tailed Student's t-test: **P < 0.005.

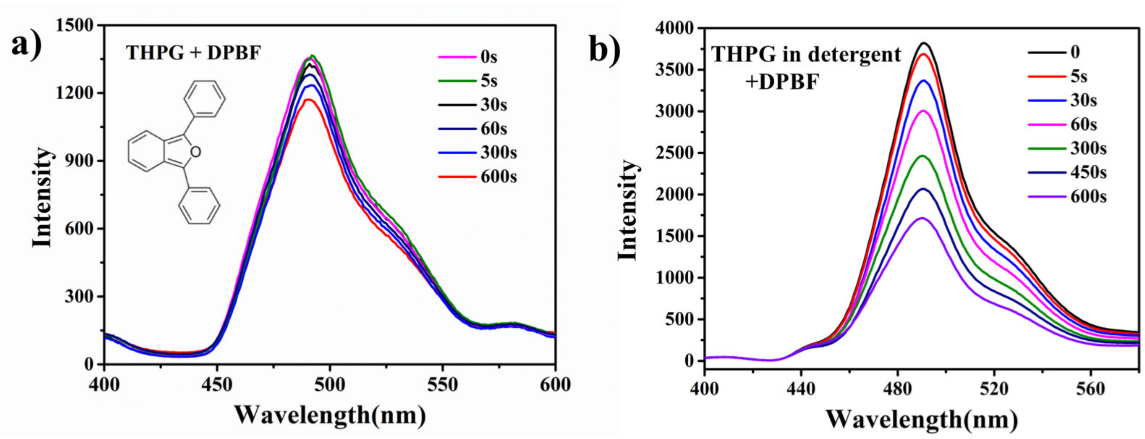

Supplementary Figure 14. Fluorescence spectra of DPBF in presence of THPG vesicle in PBS solution (a) and in 5 % Triton X-100 solution (adding 1 % DMF to dissolve DPBF) under 635 nm laser light irradiation (200 mW cm^{-2}) as a function of different time. The generation of singlet oxygen ($^1O_2$) was evaluated by monitoring the fluorescent intensity of 1,3-diphenylisobenzofuran (DPBF), which will become 1,2-dibenzoylbenzene in the presence of $^1O_2$, and thus the fluorescence intensity of DPBF will decrease[3,4]. The fluorescence intensity of DPBF in the presence of THPG vesicles in PBS solution only slightly decreased under irradiation using 635 nm laser light (200 mW cm^{-2}) for 10 min (Supplementary Figure 14a). However, as showed in Supplementary Figure 14b, at the same irradiation condition, the fluorescent intensity of DPBF reduced up to 61.2 % in THPG vesicle solution with 5% Triton X-100, in which THPG vesicles were destroyed and the FL was recovered (Figure 2i and 4a in main text). It suggested that the photodynamic ability of THPG vesicles was significantly inhibited due to the strong π-π stacking between porphyrins in the vesicles, although it is not totally inhibited. However, after the disassembly of THPG vesicles into polymers, the photodynamic ability was significantly recovered.

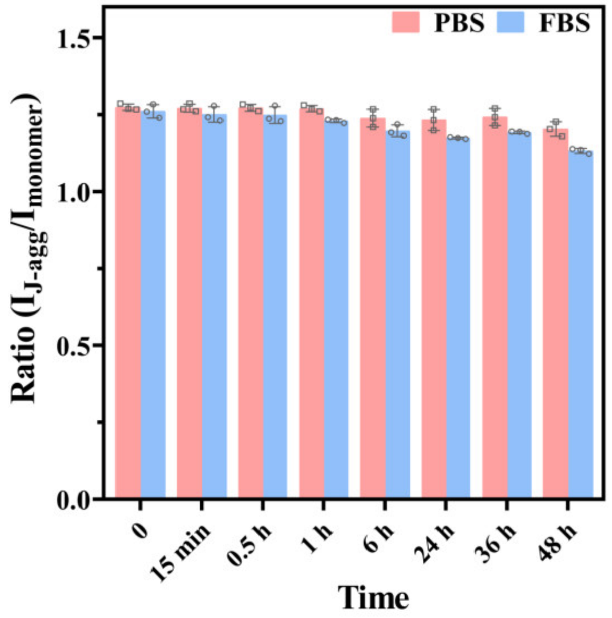

Supplementary Figure 15. Stability of THPG vesicles in PBS and PBS + 10% Fetal bovine serum (FBS) over the course of 48 h. The stability is evaluated by the absorbance intensity ratio between the peak of J-aggregation of THPG vesicles (463 nm) and THPG unimers (425 nm) (n=3 independent PBS or FBS solutions). Data are presented as mean values +/- S.D.

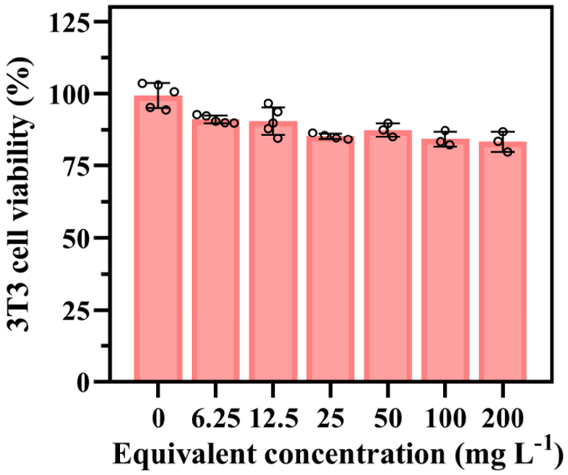

Supplementary Figure 16. Relative viabilities of 3T3 cells after being incubated with various concentrations of THPG vesicles (n=5 independent cells). Data are presented as mean values +/- S.D.

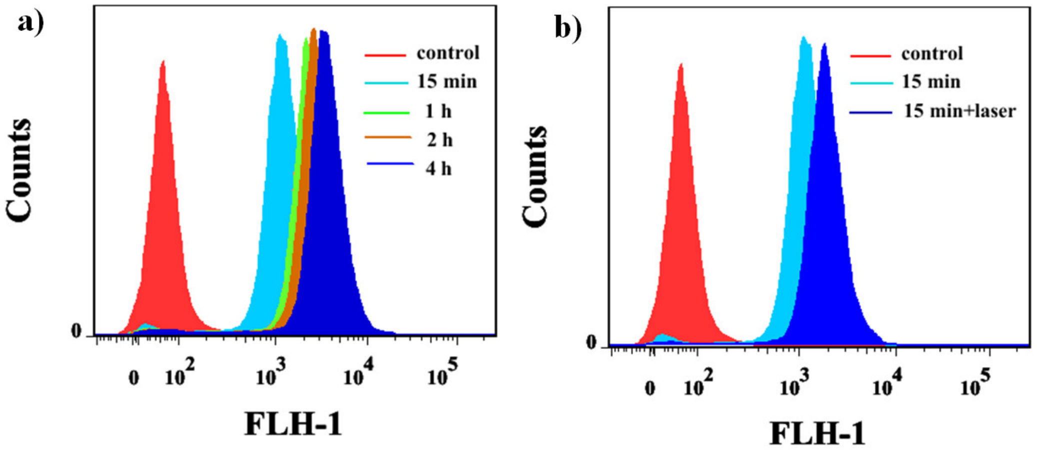

Supplementary Figure 17. a) Cellular uptake of THPG-Rb vesicles by MCF-7 cells versus the incubation time by flow cytometry analysis, the control group is blank cells. b) Cellular uptake of Rb-loaded THPG vesicles by MCF-7 cancer cells versus the incubation time with or without 635 nm laser irradiation at 50 mW cm^{-2} for 10 min by flow cytometry analysis.

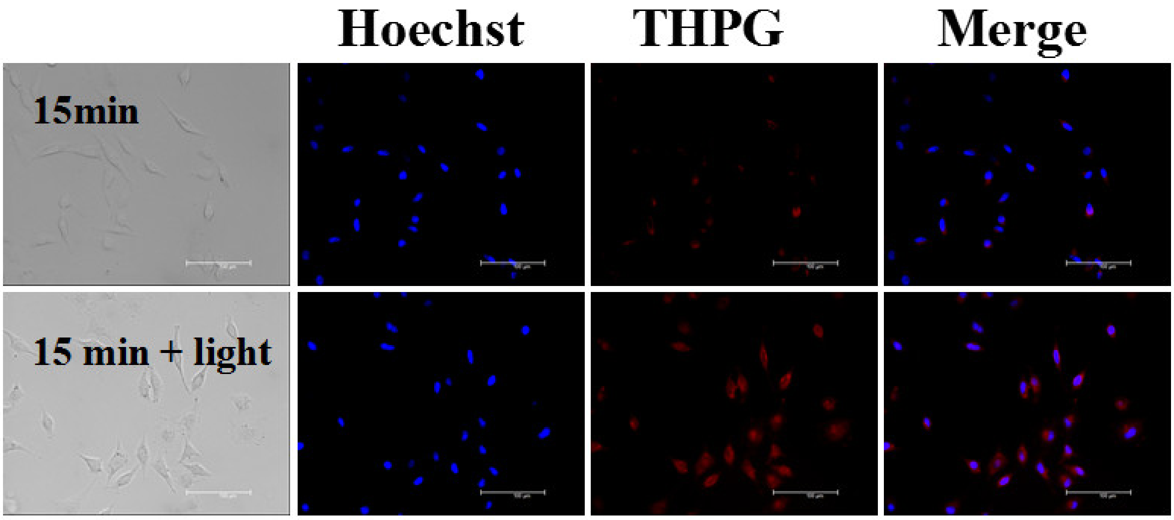

Supplementary Figure 18. Representative CLSM images of cell uptake of THPG-Rb vesicles by MCF-7 cancer cells after 15 min treatment without (up) or with (down) light exposure. The power of light laser in this experiment is 50 mW cm^{-2}. The red color is attributed to the Rb-loaded THPG vesicles and the blue color is from Hoechst. The scale bar is 100 μm. Similar images were obtained for more than 2 times experiments.

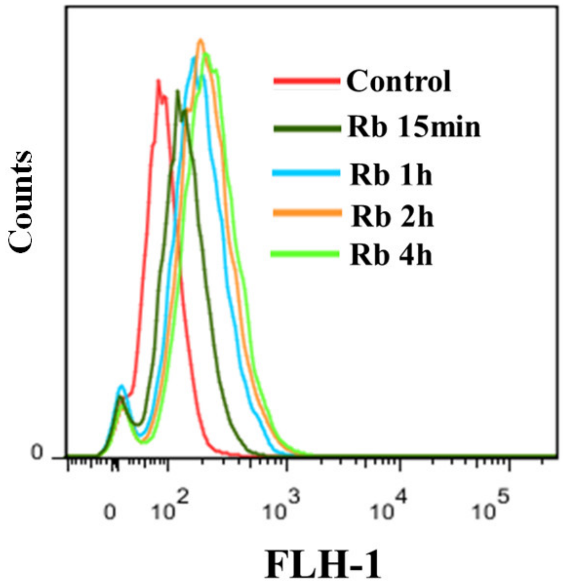

Supplementary Figure 19. Cellular uptake of free Rbs by MCF-7 cells versus the incubation time by flow cytometry analysis under different time points.

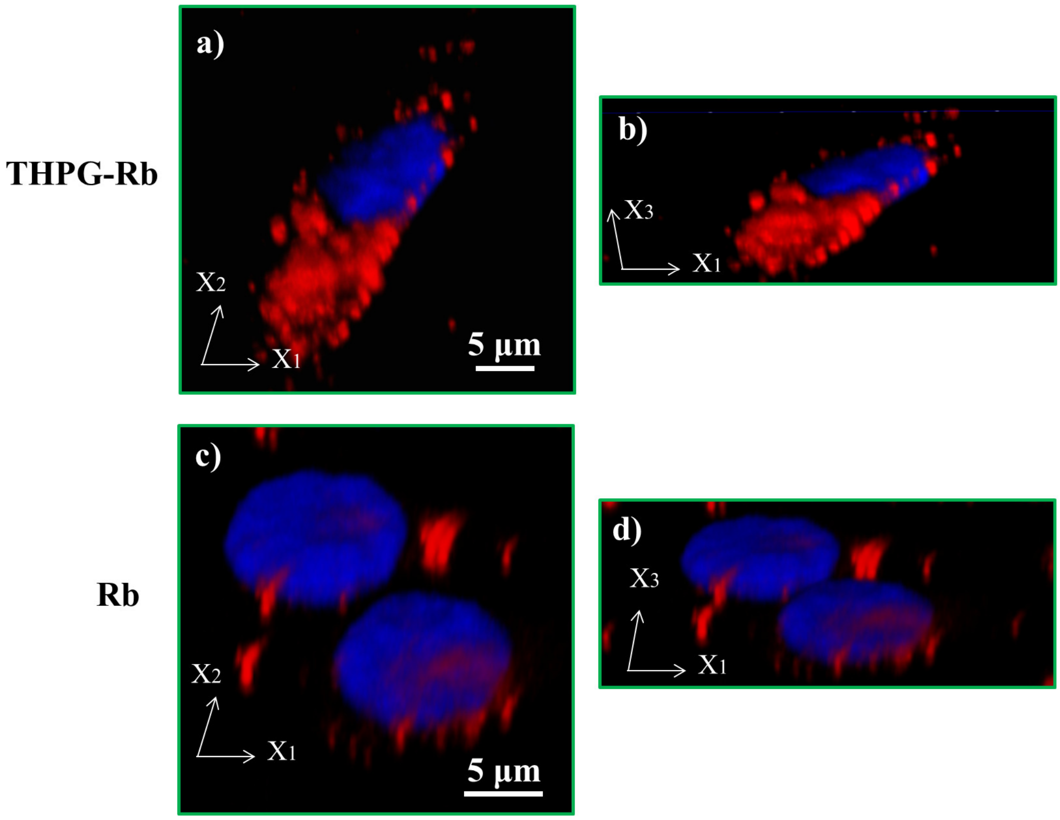

Supplementary Figure 20. Representative 3D CLSM images of MCF-7 cell incubated by THPG-Rb vesicle (a, b) and free Rb (c, d), respectively (incubation time: 2 h).

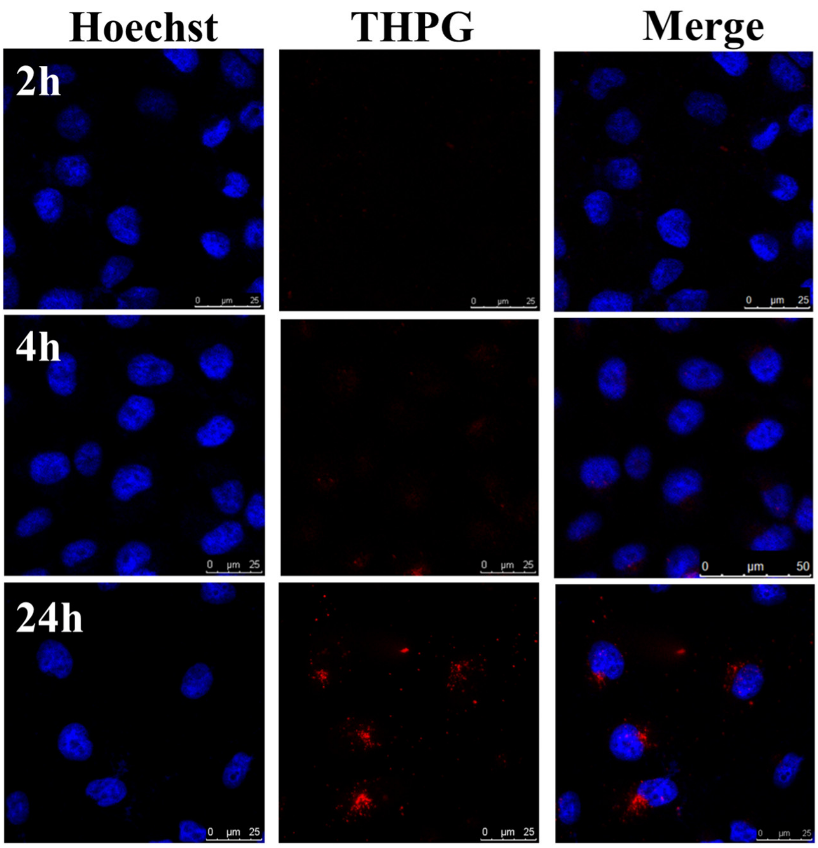

Supplementary Figure 21. The representative CLSM images of MCF-7 cancer cells internalized with THPG vesicles at various time points of 2 h, 4 h and 24 h. The red color originates from THPG vesicles after disassembly and the blue color is from Hoechst.

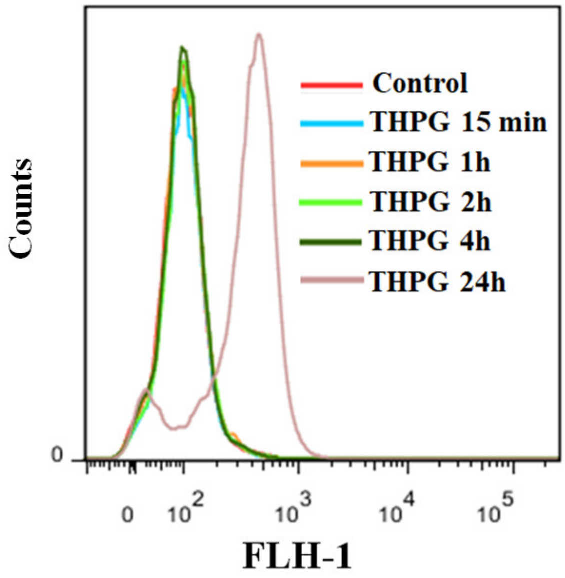

Supplementary Figure 22. Cellular uptake of THPG vesicles by MCF-7 cells versus the incubation time by flow cytometry analysis under different time points.

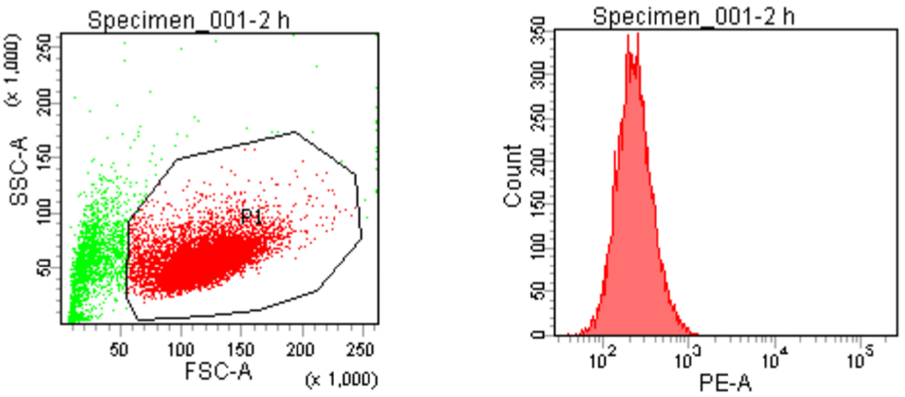

Supplementary Figure 23. Example for the gating strategy of MCF-7 cells incubated with Rb at 2 h in flow cytometry.

3. Supplementary References

1. Frisch, M., et al. Gaussian 09, revision D. 01; Gaussian, Inc. Wallingford CT (2013).
2. Grimme, S. Semiempirical GGA-type density functional constructed with a long-range dispersion correction. *Comput. Chem.* **27**, 1787 (2006).
3. Wang, S. et al. Single continuous wave laser induced photodynamic/plasmonic photothermal therapy using photosensitizer-functionalized gold nanostars. *Adv. Mater.* **25**, 3055 (2013).
4. Zhu, H. et al. Supramolecular peptide constructed by molecular Lego allowing programmable self-assembly for photodynamic therapy. *Nat. Commun.* **10**, 2412 (2019).
