# Supplementary material for: In situ supramolecular polymerization-enhanced self-assembly of polymer vesicles for highly efficient photothermal therapy
Source: Nat Commun. 2020 Apr 7;11:1724. doi: 10.1038/s41467-020-15427-1 (PMC7138818; doi:10.1038/s41467-020-15427-1)
Supplement: Supplementary file 2 — Reporting Summary [file 41467_2020_15427_MOESM2_ESM.pdf]

nature research

Corresponding author(s): Yongfeng Zhou

Last updated by author(s): Mar 2, 2020

Reporting Summary

Nature Research wishes to improve the reproducibility of the work that we publish. This form provides structure for consistency and transparency in reporting. For further information on Nature Research policies, see Authors & Referees and the Editorial Policy Checklist .

Statistics

For all statistical analyses, confirm that the following items are present in the figure legend, table legend, main text, or Methods section.

n/a	Confirmed	
☐	☒	The exact sample size (*n*) for each experimental group/condition, given as a discrete number and unit of measurement
☐	☒	A statement on whether measurements were taken from distinct samples or whether the same sample was measured repeatedly
☐	☒	The statistical test(s) used AND whether they are one- or two-sided<br>*Only common tests should be described solely by name; describe more complex techniques in the Methods section.*
☐	☒	A description of all covariates tested
☐	☒	A description of any assumptions or corrections, such as tests of normality and adjustment for multiple comparisons
☐	☒	A full description of the statistical parameters including central tendency (e.g. means) or other basic estimates (e.g. regression coefficient) AND variation (e.g. standard deviation) or associated estimates of uncertainty (e.g. confidence intervals)
☐	☒	For null hypothesis testing, the test statistic (e.g. *F*, *t*, *r*) with confidence intervals, effect sizes, degrees of freedom and *P* value noted<br>*Give P values as exact values whenever suitable.*
☒	☐	For Bayesian analysis, information on the choice of priors and Markov chain Monte Carlo settings
☒	☐	For hierarchical and complex designs, identification of the appropriate level for tests and full reporting of outcomes
☒	☐	Estimates of effect sizes (e.g. Cohen's *d*, Pearson's *r*), indicating how they were calculated

Our web collection on statistics for biologists contains articles on many of the points above.

Software and code

Policy information about availability of computer code

Data collection	GROMACS package (Version 4.5.4), Cell Quest software,Digital Micrograph, UV-Solution 2.0., RF 600, MestReNova 12.0, LAS X, IVIS® Lumina III
Data analysis	GraphPad Prism 8, origin 9.0, Excel

For manuscripts utilizing custom algorithms or software that are central to the research but not yet described in published literature, software must be made available to editors/reviewers. We strongly encourage code deposition in a community repository (e.g. GitHub). See the Nature Research guidelines for submitting code & software for further information.

Data

Policy information about availability of data

All manuscripts must include a data availability statement. This statement should provide the following information, where applicable:

- Accession codes, unique identifiers, or web links for publicly available datasets
- A list of figures that have associated raw data
- A description of any restrictions on data availability

The authors declare that the main data supporting the findings of this study are available within the article and its Supplementary Information files. Extra data are available from the corresponding author upon request.

Field-specific reporting

Please select the one below that is the best fit for your research. If you are not sure, read the appropriate sections before making your selection.

Life sciences study design

All studies must disclose on these points even when the disclosure is negative.

Sample size	Sample size was determined based on the minimum allowable error number of cells/animals, which was detailed in the method in manuscript and supporting information.
Data exclusions	No data was excluded from the calculations unless the samples varied sharply.
Replication	Number of repeats are indicated in the figure legends.
Randomization	Described in "Methods". The cells were randomly seeded in a 96-well/12-well or 6 well plates and animals were randomly divided into different groups.
Blinding	The investigators were blinded to group allocation during data collection and/or analysis.

Reporting for specific materials, systems and methods

We require information from authors about some types of materials, experimental systems and methods used in many studies. Here, indicate whether each material, system or method listed is relevant to your study. If you are not sure if a list item applies to your research, read the appropriate section before selecting a response.

Materials & experimental systems

n/a	Involved in the study	
☒	☐	Antibodies
☐	☒	Eukaryotic cell lines
☒	☐	Palaeontology
☐	☒	Animals and other organisms
☒	☐	Human research participants
☒	☐	Clinical data

Methods

n/a	Involved in the study	
☒	☐	ChIP-seq
☐	☒	Flow cytometry
☒	☐	MRI-based neuroimaging

Eukaryotic cell lines

Policy information about cell lines

Cell line source(s)	Mouse fibroblast NIH/3T3 cell and human breast carcinoma cell lines MCF-7 (Shanghai Institute of Cell Biology, the Chinese Academy of Sciences)
Authentication	Authenticated by STR.
Mycoplasma contamination	Tested nagative.
Commonly misidentified lines (See ICLAC register)	no commonly misidentified cell lines were used in the study.

Animals and other organisms

Policy information about studies involving animals; ARRIVE guidelines recommended for reporting animal research

Laboratory animals	SD rats (~200 g) and female nude mice (5-6 weeks), average body weight ca. 20 g
Wild animals	Not involved
Field-collected samples	Not involved
Ethics oversight	Approved by the ethics committee of Shanghai Jiao Tong University

Note that full information on the approval of the study protocol must also be provided in the manuscript.

Flow Cytometry

Plots

Confirm that:

- [x] The axis labels state the marker and fluorochrome used (e.g. CD4-FITC).
- [x] The axis scales are clearly visible. Include numbers along axes only for bottom left plot of group (a 'group' is an analysis of identical markers).
- [x] All plots are contour plots with outliers or pseudocolor plots.
- [x] A numerical value for number of cells or percentage (with statistics) is provided.

Methodology

Sample preparation	MCF-7 cells were seeded at a density of 3 × 105 cells/well in 6-well cell culture plates for 24 h.
Instrument	BD FACSCalibur, BD Bioscience
Software	Cell Quest AND Flowjo 7.6.1
Cell population abundance	Data for 1.0 × 104 gated events were collected.
Gating strategy	Data for 1.0 × 104 gated events were collected.

- [x] Tick this box to confirm that a figure exemplifying the gating strategy is provided in the Supplementary Information.
